# Lymph Node Metastasis-Associated Spatiotemporal Mapping of the TFF3-Linked Niche in Breast Cancer: Integrating Radiogenomic Signatures with Immune-Ecosystem Remodeling

**DOI:** 10.34133/research.1016

**Published:** 2026-01-15

**Authors:** Dianqi Cai, Chao Zhu, Haoxuan Huang, Yuchen Cao, Gengxi Cai, Zijun Chen, Junjie Feng, Weiqi Zhang, Wenjun Mao, Jianguo Lai

**Affiliations:** ^1^ Guangdong Cardiovascular Institute, Guangdong Provincial People’s Hospital (Guangdong Academy of Medical Sciences), Southern Medical University, Guangzhou 510080, Guangdong, China.; ^2^ Guangdong Provincial People’s Hospital Ganzhou Hospital, Ganzhou Municipal Hospital, Ganzhou Innovation Center, National Regional Medical Center, Gannan Medical University, Ganzhou, China.; ^3^ The First Affiliated Hospital of Nanchang University, Nanchang, Jiangxi, China.; ^4^ The Third Affiliated Hospital of Nanchang University, Nanchang, Jiangxi, China.; ^5^ Plastic Surgery Hospital, Peking Union Medical College, Chinese Academy of Medical Sciences, Beijing 100144, China.; ^6^ The First People’s Hospital of Foshan, Foshan 528000, Guangdong, China.; ^7^ Department of Thoracic Surgery, The Affiliated Wuxi People’s Hospital of Nanjing Medical University, Wuxi People’s Hospital, Wuxi Medical Center, Nanjing Medical University, Wuxi 214023, China.; ^8^ Wuxi College of Clinical Medicine, Nanjing Medical University, Wuxi 214023, China.; ^9^ Zhujiang Hospital, Southern Medical University, Guangzhou, China.

## Abstract

Primary breast cancer (PBC) with axillary lymph node metastasis (ALNM+) is associated with distinct clinical outcomes, including reduced survival (The Cancer Genome Atlas/Foshan cohorts, *P* < 0.05) and an attenuated response to anti-programmed cell death protein 1 antibody/anti-programmed death-ligand 1 antibody (anti-PD-1/anti-PD-L1) therapy. Through ALNM-stratified single-cell RNA sequencing profiling, we identified 3 hallmark immune subsets in ALNM+ PBC: (a) proliferative *MKI67*+ T cells, (b) exhausted *GZMA*+ *CD8*+ T cells, and (c) *CCL13*/*CXCL10*/*TOP2A*+ macrophages. Cross-modal integration of metastasis–epithelial–mesenchymal transition (EMT) signatures with Mendelian colocalization analysis prioritized *TFF3* as a central mechanistic regulator. We validated malignant-cell-specific *TFF3* expression across pan-cancer single-cell profiles and in PBC lineages. Integration of Mendelian colocalization signatures with pan-cancer spatial atlases established the *TFF3* oncogene as a regulator of spatial EMT programs. Radiogenomic modeling that incorporated machine-learning-derived computed tomography features identified a *TFF3*-based radiomics risk score. Spatial multi-omics analyses—including bulk RNA sequencing, proteomics, and spatial transcriptomics—established a correlation between *TFF3* expression and both MAPK signaling activation and EMT markers. Functional validation demonstrated that *TFF3* plays a dual role as an amplifier of the MAPK–EMT axis and a modulator of immune checkpoints. Critically, the prometastatic phenotype driven by *TFF3* was rescued upon pharmacological inhibition of MAPK signaling, providing direct evidence of this mechanistic link. In vivo xenograft models confirmed that *TFF3* knockdown suppressed metastasis. Pharmacogenomic screening identified 6-mercaptopurine as a novel *TFF3* antagonist, which exhibited dose-dependent inhibition of the MAPK–EMT axis. Furthermore, the antimigratory effect of 6-mercaptopurine was reversed by *TFF3* overexpression, confirming the functional specificity of this drug–target interaction. Notably, tumors with high *TFF3* expression (*TFF3*hi) exhibited elevated resistance to PD-1 inhibitors but heightened sensitivity to MAPK inhibitors, suggesting a potential theranostic framework for ALNM stratification.

## Introduction

Primary breast cancer (PBC) is the most common malignancy worldwide and accounts for approximately 30% of all cancers in women [1–5]. Advances in therapeutic strategies have substantially improved cure rates for nonmetastatic PBC. Axillary lymph node metastasis (ALNM) is a critical determinant for treatment planning and prognostic evaluation [6,7]. Axillary lymph nodes (LNs) are the primary site for PBC metastasis and the first locus to evade host immune surveillance [8,9]. However, the tumor heterogeneity between ALNM-positive (ALNM+) and ALNM-negative (ALNM−) PBC remains poorly understood. Consequently, treatment strategies often remain similar regardless of ALNM status. Despite similar approaches, patients with ALNM+ PBC have poorer survival outcomes than those with ALNM− PBC [10]. Moreover, patients with unresectable, locally advanced ALNM+ PBC show lower response rates to immune checkpoint blockade (ICB) therapy than their ALNM− counterparts [11]. Thus, a multidimensional investigation of the molecular mechanisms driving ALNM in PBC is essential to identify novel therapeutic targets and develop effective strategies.

Tumor development is driven by dynamic interactions between malignant cells and the immune microenvironment [12]. High-resolution technologies are essential for characterizing the complex heterogeneity of the tumor immune microenvironment. However, conventional bulk RNA sequencing (RNA-seq) offers limited resolution for analyzing deeply embedded microenvironmental components. In contrast, single-cell RNA sequencing (scRNA-seq) provides high-resolution transcriptomic profiles at the single-cell level [13–19]. This technology enables precise characterization of distinct cellular subpopulations and their unique biological functions [20–25]. Therefore, scRNA-seq is effectively used to investigate the tumor immune microenvironment and heterogeneity in various solid tumors [17,19,21,26–28].

Disease pathogenesis requires multidimensional interpretation [29]. Single-factor predictions are prone to measurement errors and individual variability. Multifactor models enhance signal strength by averaging out such errors, yielding more stable and accurate predictions. Radiogenomics integrates imaging features with genomic data, providing deeper insights into disease mechanisms [5,30,31].

This study integrated spatial transcriptomics (ST), scRNA-seq, and multi-omics analyses to delineate the roles of an immunosuppressive microenvironment and tumor heterogeneity in ALNM+ PBC versus ALNM− PBC. Our findings provide mechanistic insights for developing optimized therapeutic strategies for PBC patients.

## Results

### Survival prognosis and tumor ecosystem between ALNM+ and ALNM− PBC

**Fig. 1.**
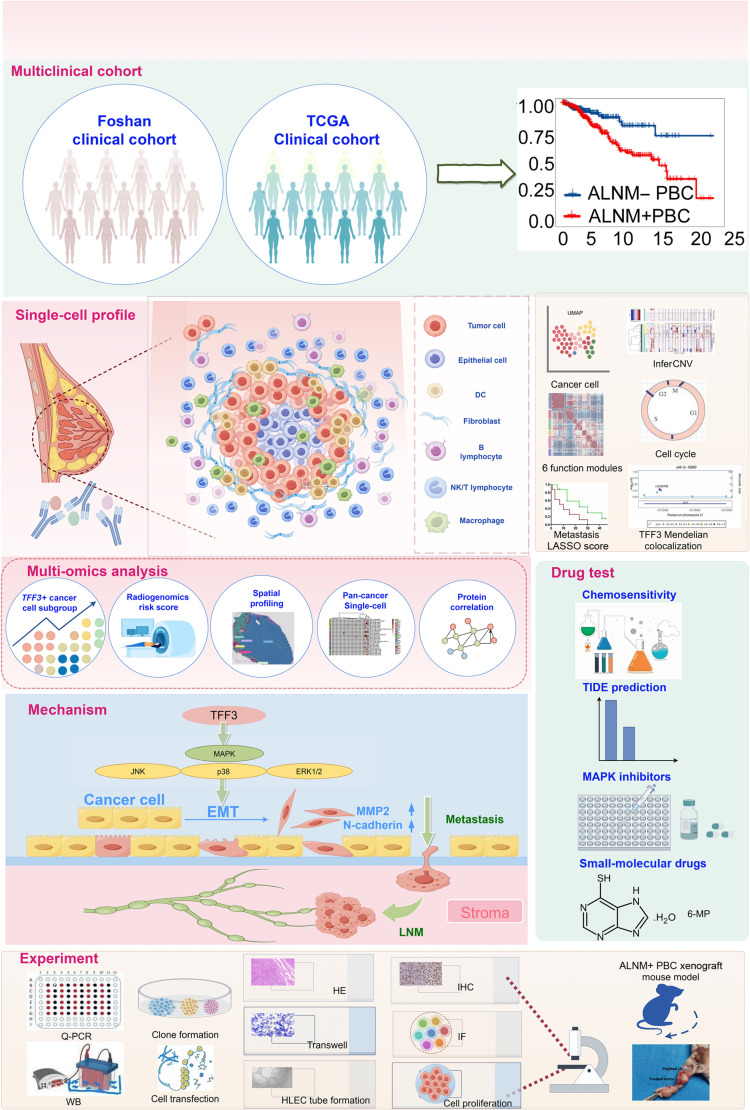
Study schematic. This study integrates single-cell insights with multi-omics datasets, including clinical cohorts, bulk RNA sequencing (RNA-seq), radiomics, proteomics, single-cell RNA-seq, and spatial transcriptomics, to investigate the molecular mechanisms and heterogeneity between primary breast cancer (PBC) patients with and without axillary lymph node metastasis (ALNM). Schematic created using Figdraw. TCGA, The Cancer Genome Atlas; OS, overall survival; PFS, progression-free survival; DFS, disease-free survival; DSS, disease-specific survival; DC, dendritic cell; NK, natural killer; UMAP, uniform manifold approximation and projection; LASSO, least absolute shrinkage and selection operator; 6-MP, 6-mercaptopurine; EMT, epithelial–mesenchymal transition; TFF3, trefoil factor 3; MAPK, mitogen-activated kinase-like protein; ERK1/2, extracellular-signal-regulated kinases 1/2; MMP2, matrix metallopeptidase 2; LNM, lymph node metastasis; Q-PCR, quantitative real-time polymerase chain reaction; WB, Western blot; HE, hematoxylin and eosin; HLEC, human lymphatic endothelial cell; IHC, immunohistochemistry; IF, immunofluorescence; LN, lymph node.

**Fig. 2.**
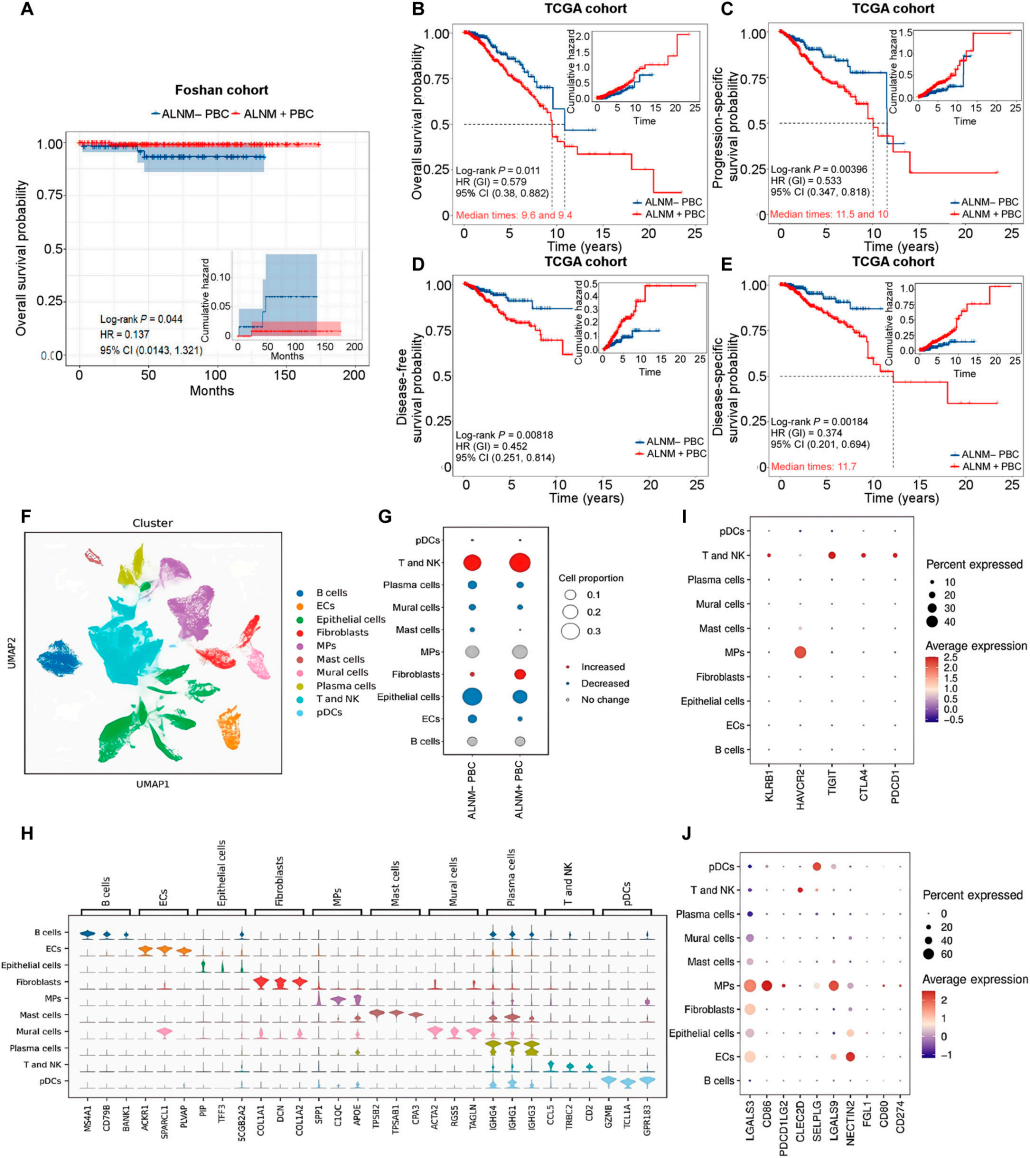
Survival prognosis and tumor ecosystem in ALNM+ and ALNM− PBC. (A to E) Survival probability of PBC with or without ALNM across 2 clinical cohorts: the Foshan cohort (A) and the TCGA cohort (B to E). The Foshan cohort represents the breast cancer clinical cohort of The First People’s Hospital of Foshan. (F to J) Single-cell RNA sequencing (scRNA-seq) samples were collected from 8 PBC cases with and without ALNM and analyzed on a genomics platform. UMAP plot of single cells from the present study, colored by major cell type (F). Changes in the proportion and number of major cell types in PBC with and without ALNM (G). Violin plot showing the expression levels of canonical marker genes in major cell types (H). Dot plots displaying the expression levels of ligand and receptor marker genes in major cell types (I and J). HR, hazard ratio; CI, confidence interval; ECs, endothelial cells; MPs, macrophages; pDCs, plasmacytoid dendritic cells.

To systematically investigate the mechanisms underlying the poor prognosis associated with ALNM in PBC, we conducted an integrated study encompassing multi-clinical cohort survival analysis, single-cell profiling of the tumor ecosystem, multi-omics exploration, and in vitro experimental validation (Fig. 1). A total of 396 PBC patients were incorporated into the radiogenomics study (Table S1). A retrospective analysis was conducted on PBC patients from the First People’s Hospital of Foshan. The results indicated that ALNM+ PBC patients had significantly poorer overall survival (OS) compared to ALNM− PBC patients (*P* < 0.05) (Fig. 2A). Moreover, based on The Cancer Genome Atlas (TCGA) database, ALNM+ PBC patients exhibited not only shorter OS (*P* < 0.05) but also reduced progression-free survival (*P* < 0.01), disease-free survival (*P* < 0.01), and disease-specific survival (*P* < 0.01) (Fig. 2B to E). These findings underscore the critical need for tailored therapeutic approaches.

To further investigate the key subsets and molecular mechanisms of PBC with ALNM, we analyzed the immune and tumor ecology differences between ALNM− and ALNM+ PBC patients using scRNA-seq. Under rigorous quality control and bimodal filtering, 85,526 cells were acquired. On average, we detected approximately 1,108 genes and 3,244 unique molecular identifiers (UMIs) per cell (Fig. S1). Unsupervised clustering analysis was then performed using the Seurat program to identify the main cell clusters with similar expression patterns (Fig. 2F and G). Each cluster was defined as a specific cell subgroup based on the expression of canonical markers and the most variable genes: B cells (markers: *MS4A1*, *CD79B*, and *CD79A*), endothelial cells (markers: *CLDN5*, *VWF*, *PECAM1*, and *CDH5*), epithelial cells (markers: *KRT18*, *KRT8*, *CDH1*, and *EPCAM*), fibroblasts (markers: *COL1A1*, *COL1A2*, and *DCN*), macrophages (MPs) (markers: *CD1C*, *C1QC*, *MRC1*, *FCN1*, and *CD14*), mast cells (markers: *CPA3*, *TPSB2*, and *TPSAB1*), mural cells (markers: *MYLK*, *TAGLN*, *MYH11*, *ACTA2*, and *RCS5*), plasma cells (markers: *IGHG1*, *MZB1*, and *JCHAIN*), T and natural killer (NK) cells (markers: *NKG7*, *TRBC2*, *TRAC*, and *CD3D*), and dendritic cells (DCs) (markers: *LILRB4*, *CLEC4C*, and *IL3RA*). Ten major cell populations were identified: B cells (*N* = 6,755), endothelial cells (*N* = 21,801), epithelial cells (*N* = 3,351), fibroblasts (*N* = 4,548), MPs (*N* = 14,053), mast cells (*N* = 877), mural cells (*N* = 2,108), plasma cells (*N* = 3,983), T and NK cells (*N* = 27,672), and DCs (*N* = 378) (Fig. 2H). The proportion of expression levels and cluster-specific markers for each cell subgroup are shown in the dot plot (Fig. 2H). To evaluate the immune microenvironment in PBC with and without ALNM, we explored the expression and correlations of immune checkpoint molecules across various cell subpopulations. We observed increased expression of *TIGIT*, *KLRB1*, *CTLA4*, and *PDCD1* (*PD-1*) in T and NK cells, with *TIGIT* showing a robust association in both cell types. Additionally, *HAVCR2* exhibited higher expression and a strong correlation in MP cells (Fig. 2I). Consistently, the immune checkpoint ligand nectin cell adhesion molecule 2 (NECTIN2) for T cell immunoreceptor with Ig and ITIM domains (TIGIT) was highly expressed in the infiltrating cells of the tumor microenvironment, while the expression of CD274 (programmed death-ligand 1 [PD-L1]) and programmed cell death 1 ligand 2 (PDCD1LG2; PD-L2), which correspond to programmed cell death 1 (PD-1), was not prominent (Fig. 2J). These results suggest that the PD-1 receptor and its ligands, PD-L1/2, may not act as independent immune checkpoint pathways in PBC with ALNM. TIGIT–NECTIN2 may represent the primary signaling pathway in PBC with ALNM. Moreover, targeting the TIGIT–NECTIN2 pathway may offer an effective treatment strategy for PBC patients with ALNM.

### Landscape of immune cells in ALNM+ and ALNM− PBC

**Fig. 3.**
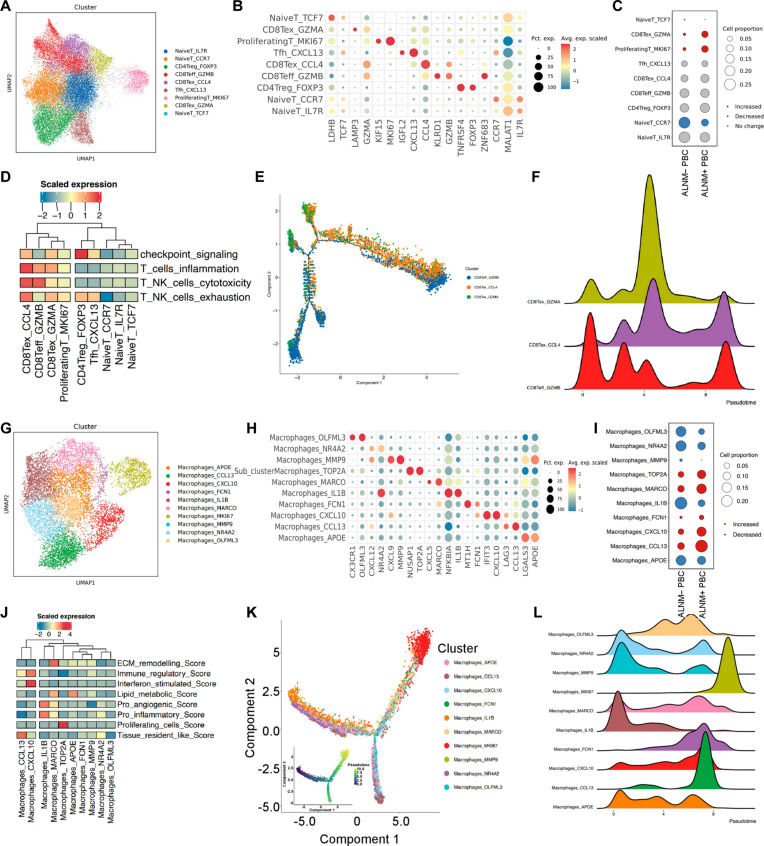
Landscape of immune cells in ALNM+ and ALNM− PBC. (A) UMAP plot showing the landscape of T cells and NK cells, colored by subcluster. (B) Dot plot showing the expression levels of canonical marker genes in each T cell and NK cell subcluster. (C) Changes in the proportion and number of each T cell and NK cell subcluster in the ALNM+ and ALNM− groups. (D) The checkpoint signaling, inflammation, cytotoxicity, and exhaustion in each T cell and NK cell cluster were analyzed and quantified based on gene signature scores. (E) Monocle pseudotime trajectory analysis of CD8+ T cell subclusters with highly variable gene expression. Each dot on the pseudotime curve represents a single cell, colored by its cluster label. (F) Differentially expressed genes and pseudotime curve of CD8+ T cell subclusters are shown in a hierarchical peak map. (G) Reclustering of macrophage cells and visualization of each cell subtype’s profile via a UMAP plot. (H) Dot plot showing the expression levels of canonical marker genes in each macrophage subcluster. (I) Changes in the proportion and number of each macrophage subcluster in groups with and without ALNM. (J) The potential biological functions and relevant signaling pathways of macrophage subclusters were evaluated using Gene Ontology (GO), hallmark, and Kyoto Encyclopedia of Genes and Genomes (KEGG) analyses. (K) Monocle pseudotime trajectory analysis of macrophages with highly variable gene expression. (L) Differentially expressed genes and pseudotime curve of macrophages are shown in a hierarchical peak map. Pct. exp., percent expressed; Avg. exp., average expression.

To elucidate the immunosuppressive landscape of ALNM in PBC, we systematically reaggregated lymphatic and myeloid immune cells for high-resolution subtype characterization. Nine T cell and NK cell subpopulations were identified in lymphatic immune cells across all samples (Fig. 3A). The expression of typical markers in each T cell subtype is depicted in the dot plot (Fig. 3B). We showed that the infiltration of naive T cells was lower in PBC with ALNM+ compared to that in ALNM− PBC, while proliferating T cells and *CD8*+ T cell exhaustion (Tex) cells were more prevalent in ALNM+ PBC (Fig. 3C). Functional cluster analyses were performed to delineate the potential functions. The toxicity and exhaustion scores of *CD8*+ T cells were distinctly higher than those of other clusters, while *CD4*+ T regulatory cells had the highest immune checkpoint score (Fig. 3D). To track the evolution of *CD8*+ T cells, we used the Monocle 2 program to depict the maturation trajectory of *CD8*+ T cell subclusters (Fig. 3E). The changes in gene expression patterns during the state transitions of *CD8*+ T cell subclusters were validated (Fig. 3F and Fig. S2A and B).

MPs and DCs are key components of the myeloid immune system. MPs were classified into 10 distinct clusters. The expression of typical biomarkers in each MP subtype is shown in the dot plot (Fig. 3G and H). In PBC with ALNM, MPs expressing *CCL13*, *CXCL10*, *MARCO*, and *TOP2A* showed greater infiltration than those in PBC without ALNM. However, the proportion of MP_IL1B was higher in the ALNM− group (Fig. 3I). MP subpopulations associated with ALNM exhibited significantly higher immune regulation, interferon stimulation, cell proliferation, extracellular matrix remodeling, and tissue retention scores compared to other subpopulations. In contrast, the highest scores in ALNM− PBC were primarily for proangiogenic and pro-inflammatory functions (Fig. 3J). The functional enrichment above highlights the characteristic functions of distinct MP subpopulations. To track the evolution of MPs, we applied the Monocle 2 program to analyze the maturation trajectory of these cells (Fig. 3K). We detected changes in gene expression patterns during MP state transitions and found that the MP_*IL1B* cluster represented the earliest stage, gradually developing into branches characterized by MP_*CXCL10*, MP_*CCL3*, and MP_*MKI67* (Fig. 3L and Fig. S2C and D). DCs were divided into 4 clusters, mainly categorized as classical DCs (cDC1 and cDC2) and mature DCs. cDC1 presents exogenous antigens via major histocompatibility complex class I to induce naive *CD8*+ T cells to differentiate into cytotoxic T lymphocytes, while cDC2 activates naive *CD4*+ T cells through major histocompatibility complex class II antigen presentation and costimulation. *LAMP3*+ mature DCs represent an immunosuppressive subtype (Fig. S3A and B). The proportion of cDC1 was higher in PBC with ALNM+ compared to that in PBC without ALNM, while the proportion of cDC2 was relatively lower (Fig. S3C). cDC1 exhibited higher oxidative phosphorylation and glycolysis scores than other subgroups (Fig. S3D). Principal component analysis elucidated the evolutionary trajectories of DCs. cDC2-1 and cDC2-2 originated from 2 branches and eventually developed into natural DCs (Fig. S3E). Gene expression changes during DC subcluster state transitions were validated (Fig. S3F and G).

### Heterogeneity landscape of ALNM+ and ALNM− PBC

**Fig. 4.**
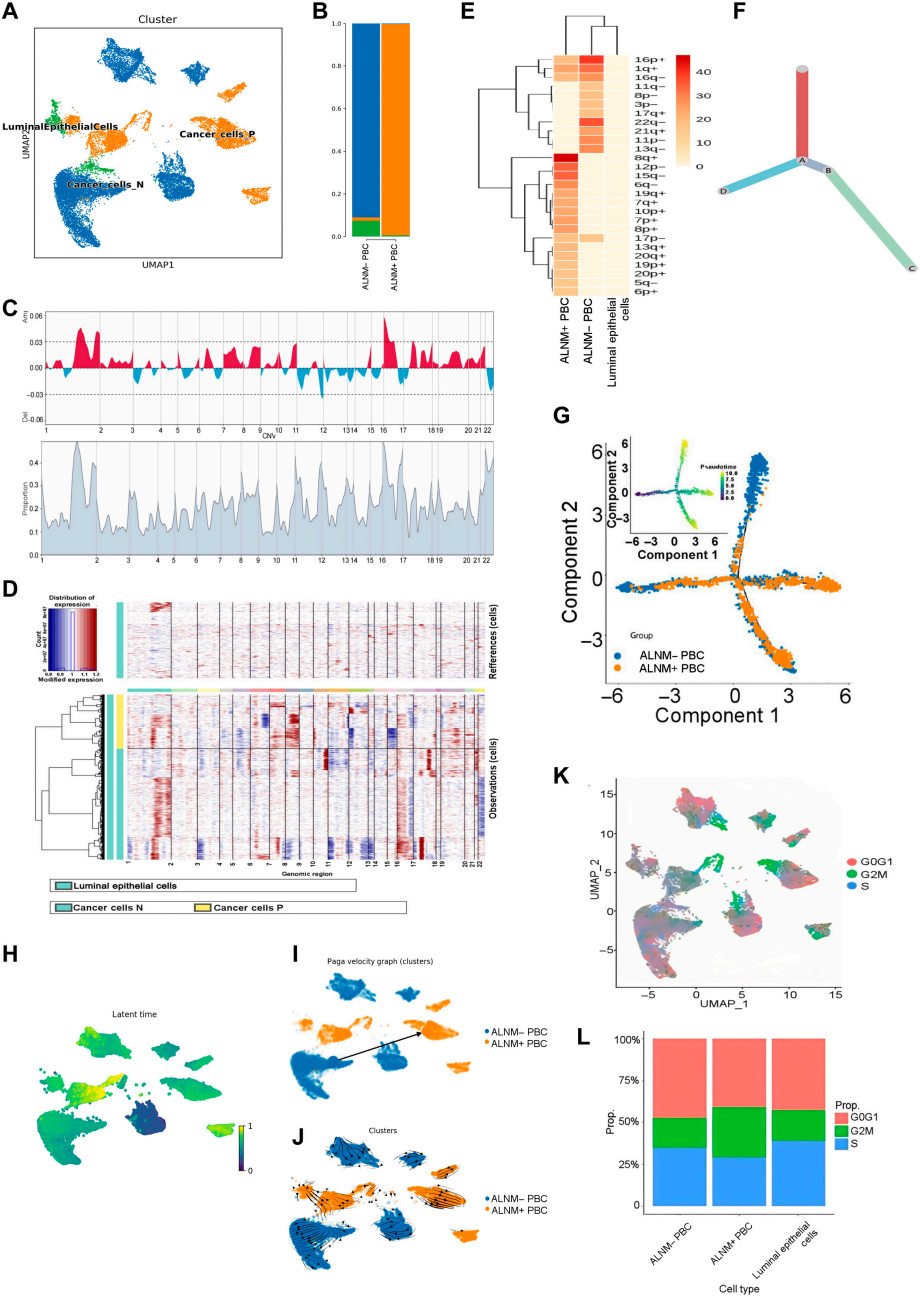
Heterogeneity landscape of ALNM+ and ALNM− PBC. (A) UMAP plot illustrating the cancer cell landscape. (B) The proportion of each cancer cell subcluster in groups with and without ALNM. (C) Hierarchical heatmap showing large-scale copy number variations (CNVs) in cancer cells and luminal epithelial cells from PBC with or without ALNM. Luminal epithelial cells were used as a control. Red represents gains; blue represents losses. (D) CNVs were inferred based on the spanning position of each chromosome (*x* axis). (E and F) Clonality trees of cancer cells in ALNM+ PBC and ALNM− PBC, with branches delineated based on the percentage of cells in subclones containing the corresponding CNVs. (G) UMAP plots displaying superimposed velocity vectors for ALNM+ PBC and ALNM− PBC, illustrating the direction and magnitude of cellular movement (represented by arrow length), thus clarifying the dynamics of individual cell migration. (H to J) Monocle pseudotime trajectory analysis of ALNM+ PBC and ALNM− PBC, focusing on highly variable gene expression. (K) UMAP plot illustrating the cancer cells in the G1, G2M, and S phases. (L) The percentage of cancer cells in the G1, G2M, and S phases within each cell cluster. The G1 phase was defined as noncycling, while the G2M and S phases were defined as cycling states. PAGA, partition-based graph abstraction.

We next analyzed transcriptome patterns and clonal evolution to characterize the heterogeneity of cancer cells. The InferCNV method was applied to differentiate tumor cells from luminal epithelial cells. We identified 19,686 malignant epithelial cells in the PBC tissues. According to differentially expressed genes (DEGs), 2 major tumor cell subpopulations were annotated (Fig. 4A). The proportional representation of each subcluster was analyzed (Fig. 4B). PBC is mainly influenced by gene copy number variations (CNVs). The inferred CNV map uncovered the heterogeneity of luminal epithelial and tumor cells in PBC with and without ALNM (Fig. 4C). Most cancer cells exhibited amplification on chromosomes 2 and 16 and deletion on chromosomes 12 and 17 (Fig. 4D). We constructed an evolutionary tree for epithelial cells based on CNVs. The tree revealed the heterogeneity in the evolutionary trajectories of subclones in ALNM+ and ALNM− PBC (Fig. 4E and F). We applied the Monocle 2 method to infer the differentiation process of cancer cells and perform pseudotime trajectory analysis in ALNM+ and ALNM− PBC (Fig. 4G). The RNA velocity method was used to further analyze the time-resolved evolution of tumor cells, investigating developmental lineage and cell dynamics (Fig. 4H to J). Notably, the ALNM− PBC subpopulation showed a trend toward ALNM+ PBC (Fig. 4I and J), suggesting that it may represent a transitional or precursor population actively transiting toward the more aggressive phenotype. In line with enhanced proliferative potential, ALNM+ PBC exhibited a prolonged G2M phase compared to ALNM− PBC, indicative of a more proliferative and mitotically active state (Fig. 4K and L).

**Fig. 5.**
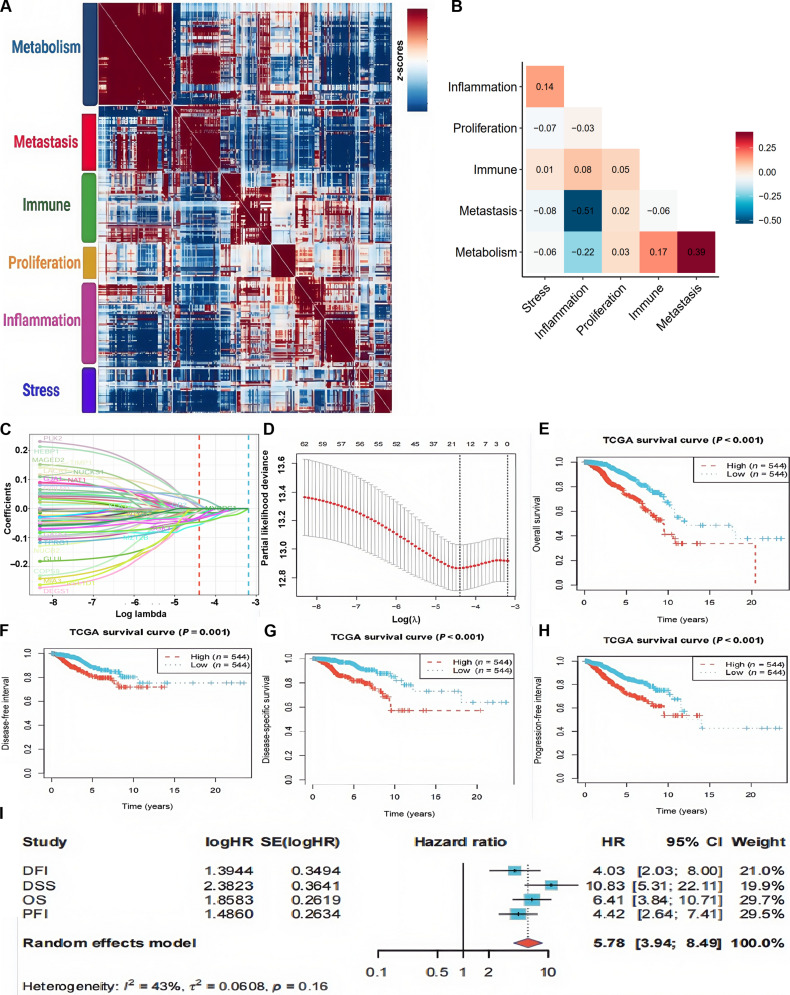
Six common expression programs in ALNM+ and ALNM− PBC. (A) A heatmap depicting pairwise correlations of 50 modules derived from the cancer cell gene expression profile. Six common expression programs (metabolism, metastasis, proliferation, inflammation, immunity, and stress) across cancer cells are clustered. (B) Correlation heatmaps showing the expression programs of 6 common tumor cell types in PBC with and without ALNM. (C) A LASSO regression coefficient path plot illustrating the relationship between the regularization parameter *λ* (lambda) and model coefficients (*y* axis), with *λ* shown on a logarithmic scale along the *x* axis. (D) Ten-fold cross-validation results visualized using a plot function, illustrating the relationship between varying *λ* (lambda) values and cross-validation errors. (E to H) A comparison of OS, PFS, DFS, and DSS between high- and low-metastasis risk scores based on The Cancer Genome Atlas Breast Invasive Carcinoma (TCGA-BRCA) cohort. (I) A comparison of OS, PFS, DFS, and DSS between high- and low-metastasis risk scores in the TCGA-BRCA cohort. DFI, disease-free interval; PFI, progression-free interval; SE, standard error.

Further, subpopulations within clusters were exploited to characterize the heterogeneity of cancer cells. We investigated 6 expression programs with distinct cell states and functions, including proliferation, metastasis, immune response, stress response, metabolism, and inflammation (Fig. 5A). Based on the scores of each program, we performed hierarchical clustering on the samples. The distribution of each expression program is shown in the map, and we conducted correlation analysis to validate pairwise interactions. This identified 3 significant co-occurrence program pairs and 2 mutually exclusive program pairs (Fig. 5B), delineating the functional interplay within the tumor cell ecosystem. To translate the most clinically relevant program into a prognostic tool, we selected characteristic genes from the metastasis program​ to construct a prognostic signature. Notably, using data from the TCGA database, we selected characteristic genes from the metastatic program to construct a least absolute shrinkage and selection operator (LASSO)-based prognostic risk model (Fig. 5C and D). Further validation across 4 clinical endpoints—OS, progression-free interval, disease-free interval, and disease-specific survival—demonstrated that higher scores were associated with poorer survival outcomes (*P* < 0.01) (Fig. 5E and H). Additionally, meta-analysis of survival risk ratios revealed a hazard ratio greater than 1, supporting a consistent conclusion (Fig. 5I).

### Joint prediction of machine learning based on radiomics feature and *TFF3* gene expression

**Fig. 6.**
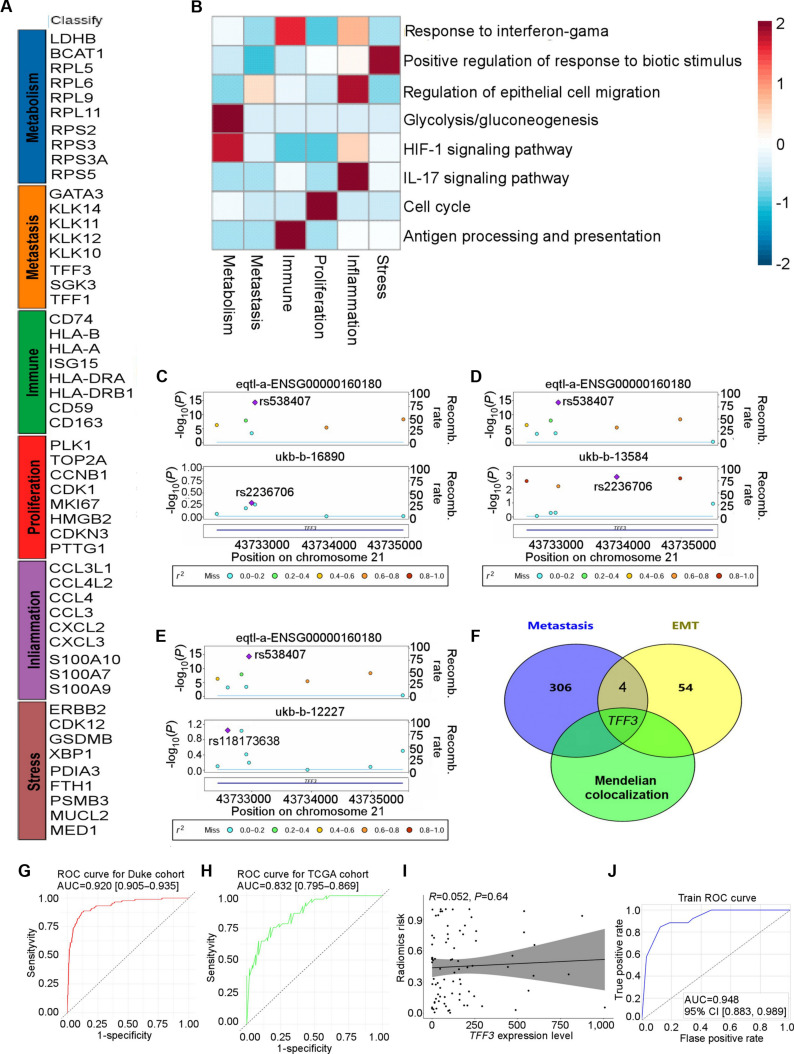
Joint prediction using machine learning based on radiomic features and *TFF3* gene expression. (A) Correlation heatmap of 6 common expression program characteristic genes in PBC with or without ALNM. (B) Gene set variation analysis (GSVA) was performed to identify functional differences across each program, with *z*-scores used for column normalization. (C to E) Mendelian colocalization analysis in PBC using the gassocplo R package for visualization. The stacking regional association maps of expression quantitative trait locus (eQTL) and genome-wide association study (GWAS) traits in genomic regions were plotted based on GRCh37(hg19) or GRCh38(hg38) coordinates. The purple diamond represents the single-nucleotide polymorphism (SNP) with the minimum *P* value corresponding to eQTL and GWAS. GWAS dataset IDs: ukb-b-16890, ukb-b-13584, and ukb-b-12227. (F) Venn diagram showing the overlap between Mendelian colocalization, the metastasis program, and the EMT gene sets. (G and H) Receiver operating characteristic (ROC) curve illustrating the predictive performance of the radiomics model in evaluating ALNM in PBC, using the Duke cohort for training and the TCGA cohort for testing. (I) Scatterplot with a fitted regression line and CIs showing the linear correlation between *TFF3* expression levels and radiomics risk in the TCGA cohort, using Pearson’s test. (J) Predictive performance of combining *TFF3* expression levels and radiomics risk using the XGBoost machine learning algorithm. HIF-1, hypoxia-inducible factor 1; IL-17, interleukin-17; AUC, area under the curve.

To characterize the heterogeneity of PBC cells, we analyzed cellular subpopulations across scRNA-seq samples. We identified 6 conserved expression programs representing distinct biological states: proliferation, metastasis, immune response, stress response, metabolism, and inflammation (Fig. 6A). Highly expressed genes within the metastasis module—including *GATA3*, *KLK14*, *KLK11*, *KLK12*, *TFF1*, *TFF3*, and *SGK3*—were notably enriched (Fig. 6A). Functional annotation revealed that the proliferation program involved cell cycle genes, immune programs were related to interferon-γ (IFN-γ) response and antigen presentation, stress programs included biological stimulus responders, metabolic programs highlighted glycolysis/gluconeogenesis and hypoxia-inducible factor 1 pathways, and inflammatory programs featured epithelial migration and interleukin-17 signaling genes (Fig. 6B). The epithelial–mesenchymal transition (EMT) score was significantly elevated in ALNM+ PBC cohorts from an independent study (Fig. S4A). We observed a positive correlation between the metastasis expression program and MAPK cascade activation in this group (Fig. S4B), which was corroborated by the finding that the MAPK signaling pathway was significantly more active in ALNM+ PBC than in ALNM− PBC (Fig. S4C).

Mendelian colocalization analysis of the 7 metastasis-associated genes across pan-cancer and breast-specific cohorts indicated that the variant rs538407 in *TFF3* exhibited a high posterior probability (PP.H4.abf > 0.75) for a shared causal genetic variant, suggesting a common mechanism across cancer types (Fig. S5A and B). This analysis confirmed a shared single-nucleotide polymorphism genetic basis between *TFF3* and PBC (Fig. 6C to E). Visualization of 3 UK Biobank breast cancer datasets—ukb-b-16890 (Fig. 6C), ukb-b-13584 (Fig. 6D), and ukb-b-12227 (Fig. 6E)—supported these findings. Integration of multi-omics and Mendelian colocalization prioritized *TFF3* as the top candidate gene. A Venn diagram illustrated the overlap between genes identified through Mendelian colocalization, the Metastasis program, and EMT-related sets (Fig. 6F).

Furthermore, we built a radiogenomics risk score (Rad-score) based on *TFF3* gene expression levels and imaging characteristics using machine learning. The model achieved an area under the curve (AUC) of 0.920 (95% confidence interval [CI] 0.905 to 0.935) in the Duke cohort (Fig. 6G) and 0.832 (95% CI 0.795 to 0.869) in the TCGA cohort (Fig. 6H). Based on these findings, we refined our predictive model by incorporating *TFF3* gene expression levels, which are known to be involved in ALNM prognosis and survival outcomes. We calculated the correlation between radiomic risk and *TFF3* expression levels using Pearson correlation and residual control scatterplot (RCS) analysis. The results showed no significant association (Pearson: *P* = 0.641, Fig. 6I; RCS: *P* = 0.918, Fig. S4D). The XGBoost model, optimized using Optuna and based on the integration of radiomic and genomic data, outperformed the radiomic model, demonstrating superior predictive ability (TCGA training: AUC = 0.948, Fig. 6J; TCGA validation: AUC = 0.873, Fig. S4E). The chi-square test confirmed the agreement in outcome distribution between the TCGA training and TCGA validation sets (*P* = 1.000), with their categorical distributions and discrepancies shown in the scatterplots and confusion matrices (Fig. S4F and G). The radiomics risk factor was a more significant contributor to the model than *TFF3* gene expression levels (Fig. S4H and I). The Kolmogorov–Smirnov test confirmed the model’s strong generalizability and reliability across datasets (Fig. S4J). The differential gene set, stratified by the Youden index, showed significant enrichment in pathways relevant to ALNM and survival prognosis, particularly the EMT and estrogen response late pathways (*P* = 0.028; *P* = 0.028), highlighting the biological relevance and predictive potential of the radiogenomics model (Fig. S4K).

### Decoding single-cell and spatial transcriptomes focusing on *TFF3*, MAPK, and EMT scores in PBC

**Fig. 7.**
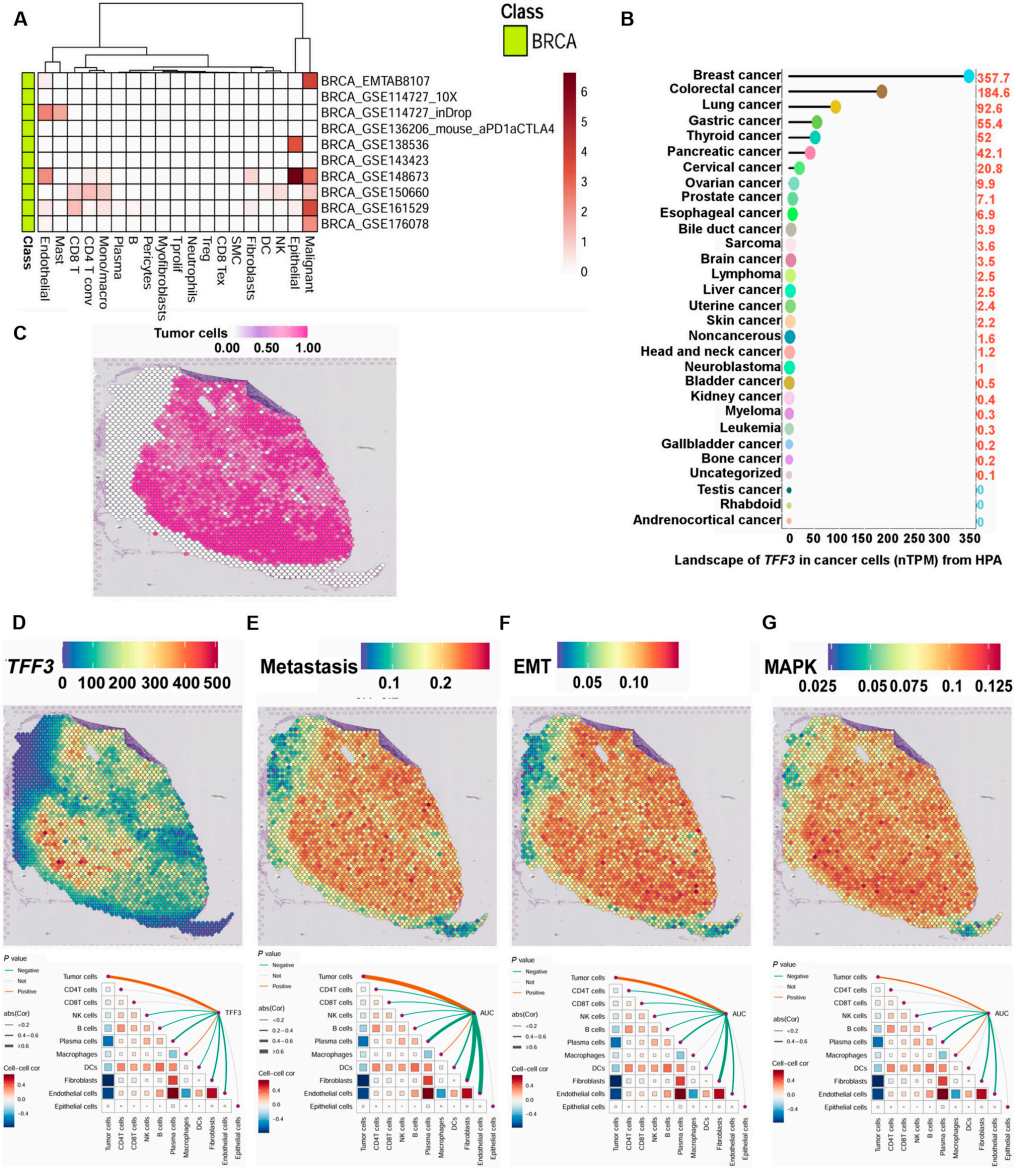
Decoding single-cell and spatial transcriptomes focusing on *TFF3*, MAPK, and EMT scores in PBC. (A) Single-cell resolution gene expression data for pan-cancer were retrieved from the Tumor Immune Single-cell Hub (TISCH) database, showing breast cancer and other tumors. Heatmaps were generated using the pheatmap package to represent the gene expression landscape. (B) The *TFF3* expression landscape in pan-cancer was obtained from the Human Protein Atlas (HPA) and Genotype-Tissue Expression (GTEx) databases. The *y* axis represents different tissues, and the *x* axis shows gene expression levels (nTPM). The positions of the points in the lollipop plot correspond to the gene expression levels across various tissues. (C to G) Spatial mapping of transcriptomes focusing on *TFF3*, metastasis expression program, MAPK cascade, and EMT scores in PBC. The Spatial Feature Plot function in the Seurat package was used to visualize the gene expression landscape in each microregion. Spearman correlation analysis was performed to assess the associations between cellular content and gene expression levels or gene scores at all spots. These correlations were visualized using the linkET package. Additional spatial features and validation from public datasets are provided in Fig. S7A to J. SMC, smooth muscle cell‌; Tex, T cell exhaustion‌; Treg, T regulatory cell; Tprolif, proliferating T cell; *CD4* T conv, conventional *CD4* T cell.

Gene expression data at a single-cell resolution for pan-cancer were acquired from the Tumor Immune Single-cell Hub (TISCH) database. To identify conserved gene expression patterns, we applied hierarchical clustering. Analysis of the pan-cancer scRNA-seq dataset deciphered that *TFF3* was predominantly expressed in malignant epithelial cells (Fig. 7A and Fig. S6A). Analysis of data from the Human Protein Atlas (HPA) and Genotype-Tissue Expression (GTEx) projects illustrated that *TFF3* expression was distinctly higher in PBC and nearly absent in immune cells (Fig. 7B and Fig. S6B).

We initially collected scRNA-seq samples (*n* = 191) from multiple specimens. Subsequently, we applied deconvolution analysis to assess the cellular composition at each point on 10x Visium slides, integrating ST with single-cell transcriptomic data. Notably, ST further confirmed that *TFF3* was primarily localized in malignant epithelial cells. Additionally, *TFF3* expression showed a positive correlation with the metastasis expression program, the MAPK signaling pathway, and the EMT score (Fig. 7C to G and Fig. S7A to J).

### *TFF3* accelerated the proliferation of BRCA cells in vivo and in vitro

**Fig. 8.**
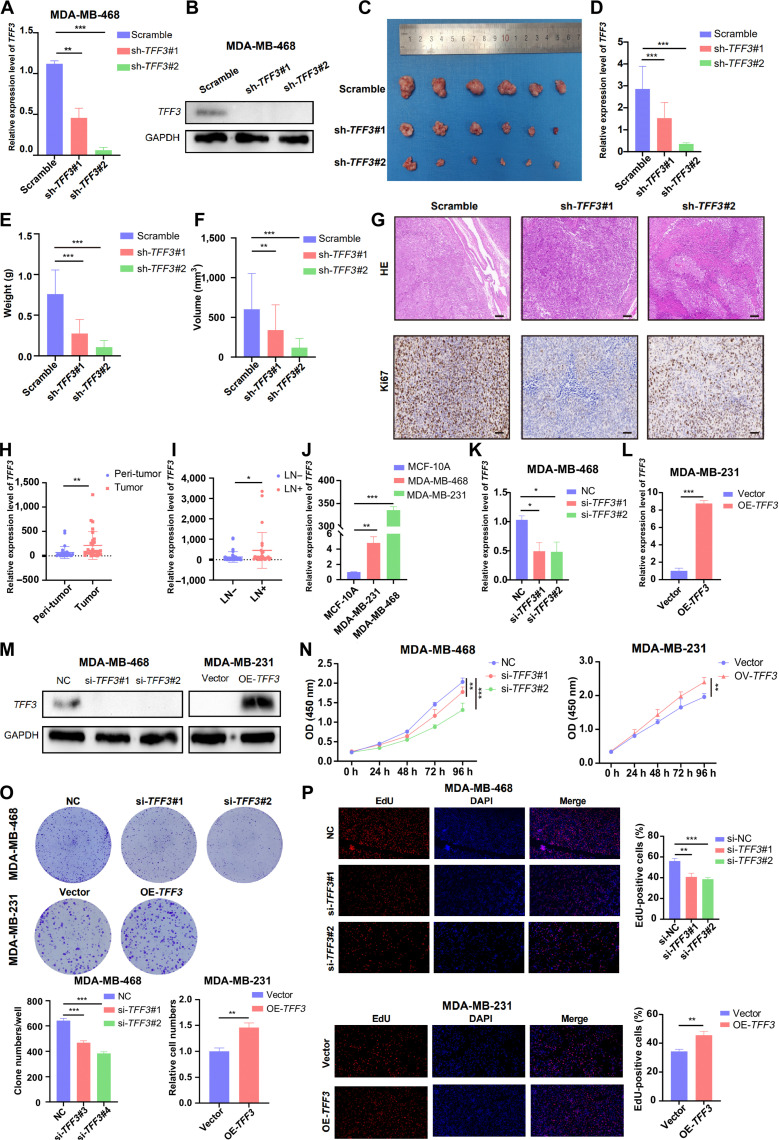
TFF3 promoted the proliferation of breast cancer cells in vivo and in vitro. (A and B) Quantitative real-time polymerase chain reaction (qRT-PCR) and Western blotting were used to confirm the construction of stable expression cell lines. MDA-MB-468 cells with TFF3 down-regulation (5 × 10^6^ cells/site) were injected subcutaneously into mice. (C) After 30 d, subcutaneous tumors were extracted from the nude mice. (D) The efficiency of TFF3 knockdown in tumors was validated by qRT-PCR. (E and F) Tumor volume was measured using a Vernier caliper, and tumor weight was measured with a balance. (G) Ki67 was used to assess tumor proliferative capacity. Scale bar: 100 μm. (H and I) TFF3 expression was higher in breast cancer tissues than in normal breast tissues and higher in ALNM+ PBC. (J) qRT-PCR was used to measure the relative expression levels of TFF3 in various breast cancer cell lines. (K to M) qRT-PCR and Western blotting were used to validate the knockdown and overexpression efficiency of TFF3. (N to P) Cell Counting Kit-8 (CCK-8), colony formation, and 5-ethynyl-2′-deoxyuridine (EdU) assays demonstrated the proliferative capacity of MDA-MB-231 and MDA-MB-468 cells after TFF3 knockdown or overexpression. *P < 0.05; **P < 0.01; ***P < 0.001. GAPDH, glyceraldehyde-3-phosphate dehydrogenase; OD, optical density; DAPI, 4′,6-diamidino-2-phenylindole.

Various omics analyses of genes were subsequently validated through experiments. A xenotransplantation model in mice was utilized to determine whether *TFF3* promotes the proliferation of BRCA (breast cancer) cells in vivo. Triple-negative breast cancer cell lines were selected for investigating lymph node metastasis in breast cancer due to several critical factors. Firstly, these cell lines exhibit a high propensity for metastasis. Secondly, there are significant treatment limitations associated with triple-negative breast cancer, as patients typically exhibit poor responsiveness to endocrine and targeted therapies, necessitating reliance on chemotherapy. Understanding the mechanisms of metastasis in this context is essential for the development of novel therapeutic strategies. Consequently, triple-negative breast cancer cell lines were chosen for the subsequent experimental investigations. First, we knocked down *TFF3* in MDA-MB-468 cells, achieving successful silencing of *TFF3* at both the messenger RNA (mRNA) and protein levels (Fig. 8A and B). MDA-MB-468 cells stably transfected with sh-*TFF3*#1, sh-*TFF3*#2, or the corresponding control sh-NC were subcutaneously inoculated into nude mice. Tumors were harvested 30 d after inoculation (Fig. 8C). Quantitative real-time polymerase chain reaction (qRT-PCR) was performed to assess the efficiency of *TFF3* knockdown in vivo (Fig. 8D). Notably, the weight and volume of tumors with *TFF3* knockdown were lower than those in the control MDA-MB-468 group (Fig. 8E and F). Finally, we performed hematoxylin and eosin (HE) staining and immunohistochemistry on the tumors and observed a decrease in Ki67 expression in tissues with *TFF3* knockdown (Fig. 8G). Overall, *TFF3* promoted the proliferation of BRCA cells in vivo. We further confirmed this result through in vitro experiments. qRT-PCR was carried out to calculate *TFF3* mRNA expression levels in PBC patients. *TFF3* mRNA expression was distinctly higher in tumor tissues than in peri-tumor tissues (Fig. 8H). To further investigate the association between *TFF3* and ALNM, we selected 34 ALNM− and 28 ALNM+ PBC patients for *TFF3* mRNA level analysis. We showcased that *TFF3* expression was higher in ALNM+ PBC patients (Fig. 8I). We then compared *TFF3* mRNA levels between 2 BRCA cell lines and 1 breast epithelial cell line. We found that *TFF3* was more highly expressed in cancer cells than in normal breast epithelial cells (Fig. 8J). Based on *TFF3* mRNA expression levels, we overexpressed *TFF3* in MDA-MB-231 cells and knocked it down in MDA-MB-468 cells. *TFF3* was successfully silenced and overexpressed at both mRNA and protein levels, as confirmed by qRT-PCR and Western blot (Fig. 8K to M). Proliferation assays, including Cell Counting Kit-8 (CCK-8), colony formation, and 5-ethynyl-2′-deoxyuridine (EdU) assays, were performed to confirm that *TFF3* increased proliferation in BRCA cells (Fig. 8N to P). Overall, *TFF3* promoted the proliferation of BRCA cells in vitro.

### *TFF3* promoted the LNM of BRCA in vitro and in vivo

**Fig. 9.**
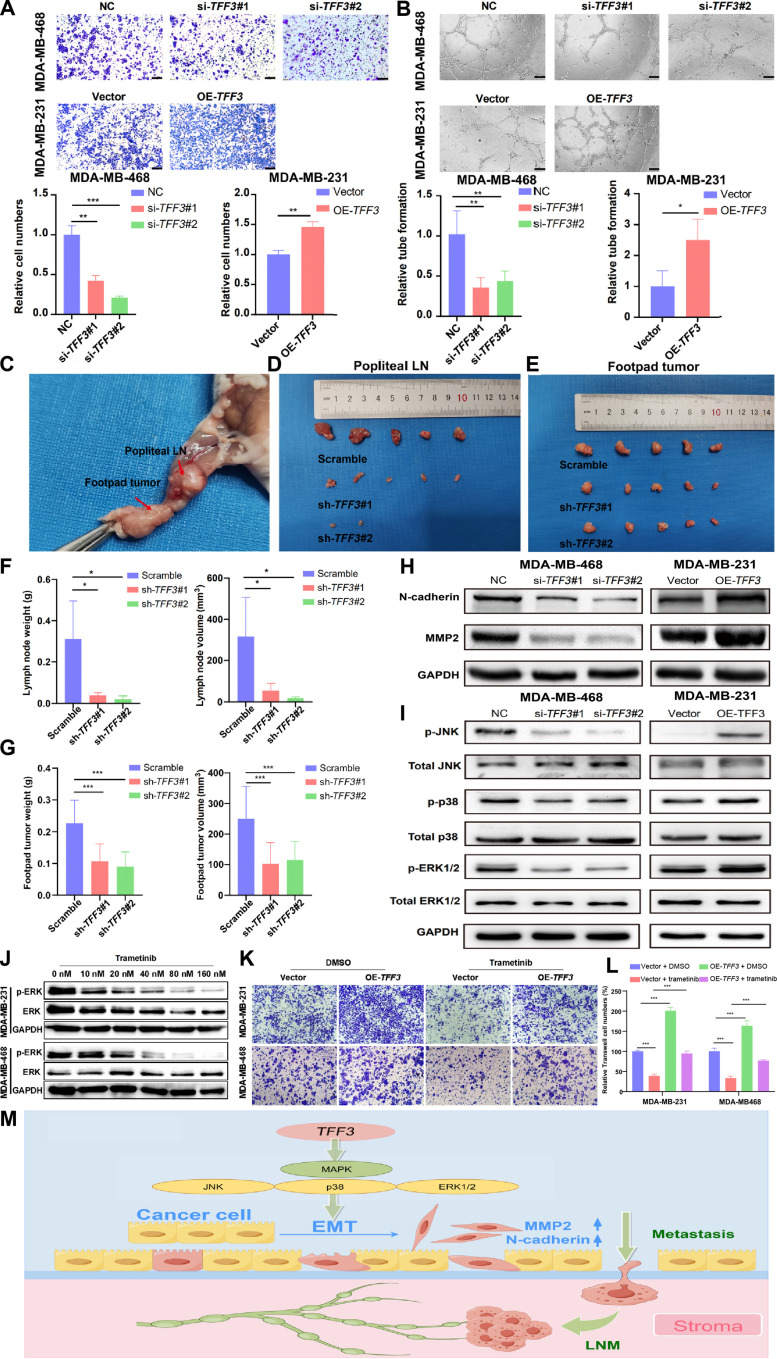
*TFF3* promotes the LNM of BRCA both in vitro and in vivo. (A) Transwell assays demonstrated the mobility of MDA-MB-231 and MDA-MB-468 cells following *TFF3* knockdown or overexpression (scale bar = 200 μm). (B) HLEC tube formation assays suggested the lymphangiogenic function of MDA-MB-231 and MDA-MB-468 cells following *TFF3* knockdown or overexpression (scale bar = 200 μm). (C) Representative images of the popliteal LNM model in nude mice, where MDA-MB-468 cells were injected into the footpads. (D and F) LN tissues were excised, and their volume and weight were measured. (E and G) Tumors were excised from the footpads, and their volume and weight were measured. (H) Western blot was used to examine the expression of EMT proteins in sh-*TFF3*-MDA-MB-468 and OE-*TFF3*-MDA-MB-468 cells. (I) Western blot was used to assess MAPK pathway protein expression in MDA-MB-468 and MDA-MB-231 cells following *TFF3* knockdown or overexpression. (J to L) Validation of MAPK pathway inhibition and functional rescue of *TFF3*-mediated metastasis. Dose-dependent decrease in p-ERK protein levels following treatment with the mitogen-activated protein kinase kinase (MEK) inhibitor trametinib in MDA-MB-231 and MDA-MB-468 cells (J). A concentration of 40 nM trametinib was selected for subsequent rescue experiments based on near-complete suppression of p-ERK expression. Rescue of *TFF3*-driven metastatic phenotype upon cotreatment with 40 nM trametinib, demonstrating dependence on MAPK pathway activation (K and L). (M) Mechanism representation of how *TFF3* promotes the LNM of BRCA. *P < 0.05; **P < 0.01; ***P < 0.001. DMSO, dimethyl sulfoxide.

The Transwell assay was performed to validate the role of *TFF3* in promoting BRCA cell migration. The results showed that *TFF3* knockdown significantly decreased the migratory ability of MDA-MB-468 cells, while overexpression of *TFF3* distinctly increased the migratory ability of MDA-MB-231 cells (Fig. 9A). A human lymphatic endothelial cell (HLEC) tube formation assay was utilized to investigate the effects of *TFF3* on lymphangiogenesis in vitro. *TFF3* knockdown weakened lymphangiogenic ability, whereas *TFF3* overexpression had the opposite effect (Fig. 9B). To further confirm that *TFF3* promotes lymph node metastasis (LNM) in BRCA cells in vivo, we performed a mouse model experiment involving footpad popliteal LNs. MDA-MB-468 cells stably transfected with sh-*TFF3*#1 and sh-*TFF3*#2, along with the corresponding control sh-NC, were injected into the footpads of mice. After 60 d, footpad tumors and LNs were harvested (Fig. 9C). The results showed that *TFF3* knockdown inhibited the weight and volume of LNs (Fig. 9D and F), and the weight and volume of footpad tumors from *TFF3* knockdown cells were lower than those of footpad tumors from control MDA-MB-468 cells (Fig. 9E and G). At the protein level, the key MAPK pathway proteins mitogen-activated protein kinase 9 (JNK2) and p38MAPK were positively correlated with *TFF3* expression (Fig. S8A). Next, we assessed the expression of metastasis-related proteins, such as matrix metallopeptidase 2 (MMP2) and N-cadherin. The results showed that *TFF3* knockdown in MDA-MB-468 cells decreased MMP2 and N-cadherin levels, whereas *TFF3* overexpression in MDA-MB-231 cells had the opposite effect (Fig. 9H). The MAPK pathway regulates cell proliferation, growth, metabolism, and apoptosis. Therefore, we studied the relationship between *TFF3* and the MAPK pathway using Western blotting. Compared to the control group, the *TFF3* knockdown group showed a distinct decrease in phosphorylated extracellular-signal-regulated kinases 1/2‌ (p-ERK1/2), p-p38, and p-JNK, while total ERK1/2, p38, and JNK levels remained unchanged. In contrast, in the *TFF3* overexpression group, p-ERK1/2, p-p38, and p-JNK levels were significantly increased, while total ERK1/2, p38, and JNK showed no significant changes (Fig. 9I). These results indicate that *TFF3* promotes BRCA proliferation and metastasis by activating MAPK signaling. In various PBC cell lines, *TFF3* gene expression is positively correlated with activation of the metastasis–EMT-up pathway. Notably, a decrease in *TFF3* expression, resulting from CRISPR knockout or methylation silencing, correlates with reduced activation of the metastasis–EMT-up pathway (Fig. S8B).

To functionally validate the role of MAPK signaling in *TFF3*-mediated metastasis, we performed rescue experiments using the mitogen-activated protein kinase kinase inhibitor trametinib. We first confirmed the efficacy of trametinib in MDA-MB-231 and MDA-MB-468 cells, where treatment with increasing concentrations led to a dose-dependent decrease in p-ERK protein levels (Fig. 9J). Based on this dose–response assay, a concentration of 40 nM trametinib, which nearly completely abolished p-ERK expression, was selected for subsequent functional rescue experiments. Importantly, the prometastatic phenotype induced by *TFF3* was effectively rescued upon cotreatment with trametinib, as demonstrated by Transwell migration (Fig. 9K and L). These results provide direct evidence that *TFF3* promotes breast cancer metastasis primarily through activation of the MAPK signaling pathway. A mechanism representation of how *TFF3* promotes the LNM of BRCA is depicted in Fig. 9M.

### 6-Mercaptopurine inhibited the proliferation and LNM of BRCA in vitro

To explore the potential of *TFF3* as a therapeutic target, we calculated the correlation between *TFF3* gene expression and the IC_50_ values of various MAPK pathway inhibitors, including JNK9L (*R* = 0.52, *P* < 0.001), CMK (*R* = 0.43, *P* < 0.001), XMD8.85 (*R* = 0.33, *P* < 0.001), PLX4720 (*R* = 0.26, *P* < 0.001), SL.0101.1 (*R* = 0.25, *P* < 0.001), and CI.1040 (*R* = 0.20, *P* < 0.001) (Fig. S8C). Differences in drug sensitivity, as measured by IC_50_ values, were evident between groups with high and low *TFF3* expressions. These results suggest that higher *TFF3* expression is associated with increased IC_50_ values and greater drug resistance (Fig. S9A). Additionally, *TFF3* gene expression was positively associated with the AUC values of chemotherapy drugs in the Genomics of Drug Sensitivity in Cancer version 1‌ (GDSC1) database, indicating that higher *TFF3* expression corresponds to lower chemotherapy sensitivity (Fig. S9B).

In further investigation of the relationship between *TFF3* and immunotherapy, we found no significant difference in ICB efficacy between the high- and low-*TFF3*-expression groups in BRCA (Fig. S10A). However, a significant difference was observed in triple-negative BRCA. Specifically, the IC_50_ of ICB treatment was higher, indicating stronger drug resistance in the high-*TFF3*-expression group (Fig. S8D). In our pan-cancer analysis of 6 immune subtypes, we observed that the C2 subtype, characterized by the dominance of IFN-γ, was distinctly lower in high-*TFF3*-expression groups than in low-*TFF3* groups. This result suggests a strong association between high *TFF3* expression and reduced IFN-γ activity (Fig. S10B). Several immune scoring metrics, including IFN-γ, methylation-based tumor-infiltrating lymphocytes (MeTIL), T cell inflamed, and cytolytic activity (CYT) scores, show a strong correlation between elevated *TFF3* expression, reduced IFN-γ, and lower immune infiltration levels (Fig. S10C to F). Our pan-cancer Pearson correlation analysis revealed a significant negative correlation between high *TFF3* expression and key immune microenvironment factors, including chemokine-related genes, chemokine receptor genes, immunoinhibitor genes, immunostimulator genes, and major histocompatibility complex genes (Fig. S10G).

**Fig. 10.**
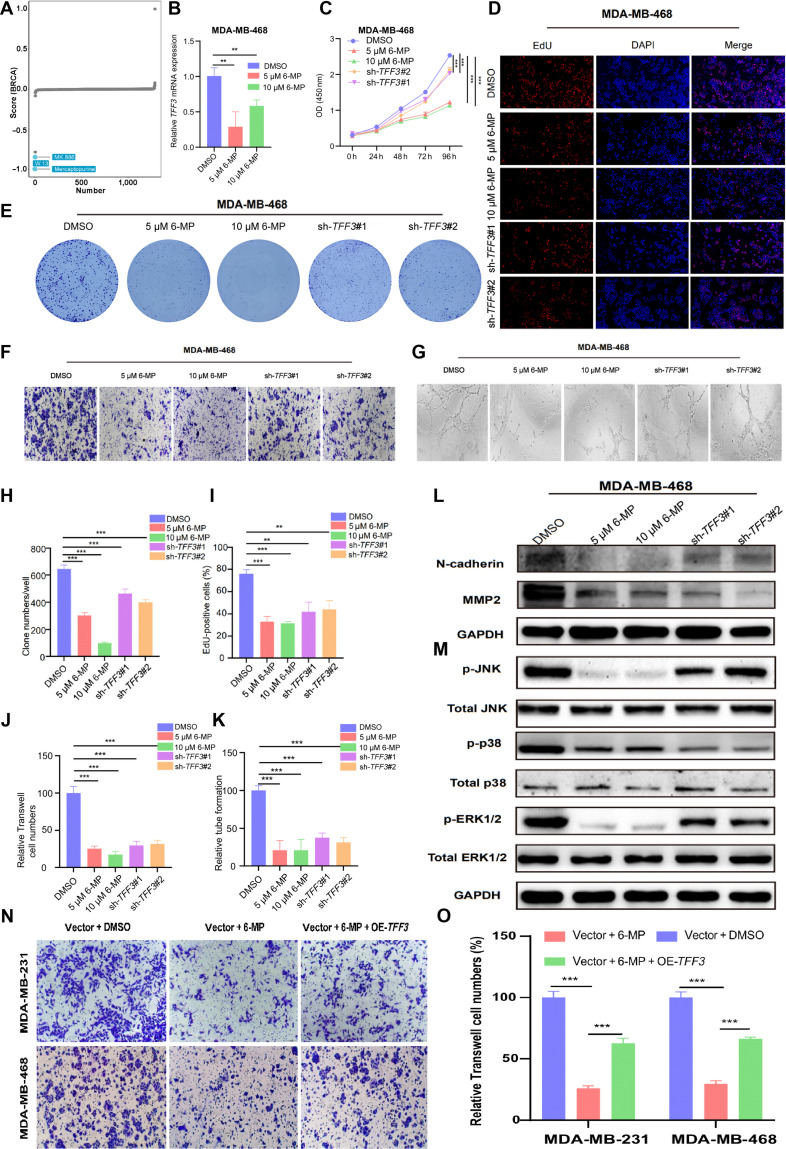
6-MP inhibited the proliferation and LNM of breast cancer (BRCA) cells in vitro. (A) The eXtreme Sum (XSum) algorithm identified potential small molecules and drugs that could correct biological effects caused by dysregulated *TFF3* gene expression in BRCA, based on data from the Connectivity Map‌ (cMAP) database. Each scatter point represents a distinct compound, with the *y* axis showing similarity scores for 1,288 compounds, derived by comparing gene-related features using the XSum method. Compounds with lower scores may inhibit gene-mediated oncogenic effects. (B) qRT-PCR was performed to assess the messenger RNA (mRNA) expression levels of *TFF3* after treatment with 5 μM/10 μM 6-MP. (C to E) CCK-8, colony formation, and EdU assays demonstrated the proliferative capacity of MDA-MB-468 cells following treatment with 5 μM/10 μM 6-MP and *TFF3* knockdown. (F) Transwell assays assessed the mobility of MDA-MB-468 cells following treatment with 5 μM/10 μM 6-MP and *TFF3* knockdown (scale = 200 μm). (G) HLEC tube formation assays demonstrated the lymphangiogenic function of MDA-MB-468 cells following treatment with 5 μM/10 μM 6-MP and *TFF3* knockdown (scale = 200 μm). (H to K) The bar charts illustrate the differences observed in colony formation (H), EdU (I), Transwell assays (J), and HLEC tube formation assays (K). (L and M) The levels of EMT proteins were examined following treatment with 5 μM/10 μM 6-MP and *TFF3* knockdown by Western blot (L). p-ERK1/2, p-p38, p-JNK, and total ERK1/2, p38, and JNK were analyzed by Western blot (M). (N and O) Rescue experiment validating the functional specificity of 6-MP targeting *TFF3*. Migration assays demonstrated that 6-MP inhibited the migration of breast cancer cells, while *TFF3* overexpression partially reversed this inhibitory effect (N). The reversal of 6-MP’s action by *TFF3* overexpression supports the functional specificity of the 6-MP–*TFF3* interaction (O). *P < 0.05; **P < 0.01; ***P < 0.001.

In the search for small molecules targeting *TFF3*, the eXtreme Sum (XSum) algorithm identified potential drugs based on dysregulated *TFF3* gene expression across various tumors, particularly in BRCA. 6-Mercaptopurine (6-MP) was identified as an effective small-molecule drug (Fig. S8E and Fig. 10A). qRT-PCR was conducted to assess *TFF3* mRNA expression levels in MDA-MB-468 cells treated with varying concentrations of 6-MP (Fig. 10B). CCK-8, colony formation, and EdU assays were performed to determine whether 6-MP reduced proliferation in MDA-MB-468 cells similarly to *TFF3* knockdown (Fig. 10C to E, H, and I). To demonstrate that both 6-MP and *TFF3* knockdown inhibit the migration of MDA-MB-468 cells, we performed Transwell assays (Fig. 10F and J). We conducted a HLEC tube formation assay to explore the effects of 6-MP on lymphangiogenesis in MDA-MB-468 cells. The results showed that 6-MP reduced lymphangiogenesis similarly to *TFF3* knockdown (Fig. 10G and K). Western blot analysis demonstrated that 6-MP reduced the levels of migration-related proteins (MMP2 and N-cadherin) in MDA-MB-468 cells, similarly to *TFF3* knockdown (Fig. 10L). Next, we analyzed the MAPK pathway. The results revealed that 6-MP decreased the levels of p-ERK1/2, p-p38, and p-JNK but had no influence on the total protein levels of ERK1/2, p38, and JNK (Fig. 10M). To assess the functional specificity of 6-MP targeting *TFF3*, a rescue experiment was performed by overexpressing *TFF3* in the presence of 6-MP. Migration assays demonstrated that 6-MP inhibited the migration of breast cancer cells. However, *TFF3* overexpression partially reversed this 6-MP-induced suppression of migration (Fig. 10N and O). The reversal of 6-MP’s effects by *TFF3* overexpression supports the functional specificity of this drug–target interaction. Overall, 6-MP inhibited the proliferation and LNM of BRCA cells in vitro.

## Discussion

ALNM+ PBC patients exhibit worse survival outcomes and lower response rates to immune checkpoint therapy compared to ALNM− PBC patients [10,11]. In this study, we conducted retrospective analyses using the TCGA and Foshan cohorts, revealing that ALNM+ PBC patients experience worse survival outcomes compared to ALNM− PBC patients. However, the tumor heterogeneity between ALNM+ PBC and ALNM− PBC remains poorly understood. Therefore, exploring the potential mechanisms of ALNM in PBC from a multidimensional perspective is crucial for achieving precise treatment targets.

To further explore the key subsets and molecular mechanisms of PBC with ALNM, we employed scRNA-seq to examine the immune and tumor ecological heterogeneity between ALNM− and ALNM+ PBC patients. We uncovered that the immune microenvironment of ALNM+ PBC was primarily characterized by *MKI67*+ proliferative T cells, *GZMA*+ *CD8*+ Tex cells, and MPs expressing *CCL13*, *CXCL10*, and *TOP2A*. Furthermore, this high-metastasis gene profile was positively correlated with MAPK pathway activation and was associated with EMT. Based on this high-metastasis gene profile, we constructed a LASSO-based risk prognostic model for BRCA. By intersecting the gene profiles associated with metastasis and EMT, we performed Mendelian colocalization analysis to show that the *TFF3* gene shares a common single-nucleotide polymorphism genetic basis with BRCA. In a pan-cancer scRNA-seq cohort, *TFF3* was predominantly expressed in malignant epithelial cells, with its peak expression observed in BRCA cell lines.

*TFF3* has been shown to facilitate malignant progression in various cancers [32–34]. *TFF3* levels are significantly elevated in colorectal cancer, where it promotes malignant progression through EMT [35]. In pituitary adenomas, vascular endothelial growth factor A promotes cancer cell migration and angiogenesis [36]. Moreover, it has been confirmed that these factors contribute to the malignant progression of various cancers [35,37,38]. We observed that high *TFF3* expression promoted lymphatic vessel formation and LNM in BRCA. Previous studies have suggested that *TFF3* is closely associated with cancer migration in various cancers, although less research has focused on its role in LNM. Therefore, we further explored the role of *TFF3* in LNM in BRCA. *TFF3* is expected to be a novel molecular target for both the treatment and prediction of LNM in BRCA.

The integration of artificial intelligence has enhanced MRI feature analysis, improving predictive accuracy while introducing challenges in interpretability [39]. Our radiogenomics model, which integrates *TFF3* expression and MRI features, demonstrated strong prognostic efficacy for predicting ALNM. Additionally, we employed bulk RNA-seq, pan-cancer single-cell transcriptomics, ST, and proteomics to show that *TFF3* is positively correlated with MAPK activation and EMT. Analysis of large BRCA cell line datasets uncovered a positive association between *TFF3* and the metastasis–EMT-up pathway. Our in vitro studies (including qRT-PCR, Western blot, CCK-8 assays, EdU assays, colony formation assays, HLEC tube formation assay, Transwell assays, small interfering RNA [siRNA] transfection, knockout experiments, HE staining, and immunohistochemistry) and in vivo xenograft models demonstrated that *TFF3* promotes EMT and LNM through MAPK pathway activation. Critically, pharmacological inhibition of MAPK signaling rescued the prometastatic phenotype driven by *TFF3*, providing direct functional validation of this mechanistic link. Furthermore, using the XSum algorithm, we identified 6-MP as a potential small molecule to correct *TFF3* dysregulation. Experiments showed that 6-MP inhibited MAPK pathway activation by reducing *TFF3* expression. Additionally, the antimigratory effect of 6-MP was reversed by *TFF3* overexpression, confirming the functional specificity of this drug–target interaction.

This study has several limitations. First, although we developed a novel radiogenomics model, its clinical utility requires prospective validation in a larger sample cohort. Second, further molecular mechanism studies are needed in the future.

In summary, this study combined spatial and single-cell transcriptomics with multi-omics analysis to elucidate the roles of the immune microenvironment and tumor heterogeneity in ALNM+ and ALNM− PBC patients (Fig. 1). We identified that the *TFF3*–MAPK–EMT axis promotes LNM, positioning *TFF3* as a therapeutic target and 6-MP as a potential treatment strategy.

## Materials and Methods

### Tissue sample

Tumor tissues were acquired from 8 patients with PBC who were undergoing surgery. Among these, 4 patients had ALNM, while the other 4 did not. None of the 8 patients had received neoadjuvant chemotherapy before surgery.

### Tissue dissociation and single cell isolation

After surgery, fresh tissues were immediately placed on ice in sCelLive Tissue Preservation Solution. The specimens were then washed 3 times with Hanks’ balanced salt solution, chopped into small pieces, and digested with 3 ml of sCelLive Tissue Dissociation Solution using the PythoN Tissue Dissociation System at 37 °C for 15 min. Red blood cells were removed by incubating the mixture with red blood cell lysis buffer at room temperature for 5 to 8 min. The supernatant was removed by centrifuging the mixture at 300 × g for 5 min at 4 °C, followed by gentle resuspension in phosphate-buffered saline (PBS).

### Single-cell transcriptome library preparation and sequencing

A microwell chip was used to capture barcoding beads, which were then employed for reverse transcription and PCR amplification. In accordance with the GEXSCOPE Single Cell RNA Library Kits protocol, scRNA-seq libraries were developed [40]. Individual libraries were built on Illumina NovaSeq 6000 using 150-bp paired-end reads.

### Primary analysis of raw read data

CeleScope was used to remove low-quality reads from the raw data, and Cutadapt v1.17 was employed to trim the poly-A tail and adapter sequences [41]. The UMI and cell barcode were extracted. The reads were then mapped to the reference genome GRCh38 using STAR v2.6.1a [42]. The featureCounts v2.0.1 program was used to obtain the UMI and gene counts for each cell, which were then used to generate expression matrices for further analysis [43].

### DEG analysis

The scanpy.tl.rank_genes_groups function was used with the Wilcoxon rank-sum test and default parameters to identify DEGs. The adjusted *P* value was calculated using the Benjamini–Hochberg correction, and statistical significance was evaluated with a threshold of 0.05.

### Cell type annotation

The expression of canonical markers identified in the DEGs through the SynEcoSys database was used to determine the cell type identity of each cluster. Seurat v3.1.2 (DoHeatmap, DotPlot, and VlnPlot) was used to generate heatmaps, dot plots, and violin plots to visualize the expression of markers distinguishing the different cell types.

### Pathway enrichment analysis

“clusterProfiler” R package v3.16.1 was used to conduct Gene Ontology and Kyoto Encyclopedia of Genes and Genomes analyses to investigate the roles of DEGs [44]. Pathways with an adjusted *P* value less than 0.05 were considered distinctly enriched.

### UCell gene set scoring

The R package UCell (v1.1.0) was used to score the gene set. UCell scores were calculated using the Mann–Whitney *U* statistic, which ranks genes based on their expression levels in specific cells. UCell’s rank-based scoring system allows its application to large datasets containing diverse samples and batches. Trajectory and switch gene analyses were performed using Monocle 2 to reconstruct the cell differentiation trajectory [45]. Cells were grouped based on their spatial and temporal differentiation using highly variable genes. DDRTree was used for feature selection (FindVariableFeatures) and dimensionality reduction. The switching point was defined as the time at which the fitted line crossed the 0.5 probability threshold for genes exhibiting a distinct bimodal “on–off” distribution.

### Functional gene module analysis (hotspot)

For module identification, the “danb” model was used to select the top 500 genes with the highest autocorrelation *z*-scores. Modules were identified using the create_modules function with parameters min_gene_threshold = 15 and fdr_threshold = 0.05. Module scores were computed using the calculate_module_scores function.

### Collection and processing of bulk transcriptome data

Bulk RNA-seq transcriptome and clinical data were retrieved from the TCGA database. Kaplan–Meier curves and the survival package in R were used to calculate *P* values via the log-rank test, comparing survival differences between high- and low-expression groups.

### Constructing a risk score model using the LASSO algorithm

The TCGA Breast Invasive Carcinoma dataset, containing clinical data from PBC patients and RNA-seq information, was used to develop a risk score model based on metastatic gene profiles. LASSO regression was conducted with the family parameter set to binomial, indicating a logistic regression model for binary classification. The nlambda parameter was set to 100, allowing glmnet to generate a sequence of *λ* values from 0 (no regularization) to the maximum threshold, totaling 100 *λ* values for model fitting. The alpha parameter was set to 1, indicating that the model uses LASSO regression with L1 regularization. The cv.glmnet function performed 10-fold cross-validation, dividing the data into 10 portions: 9 for training and 1 for validation. For cross-validation, deviance was specified as type.measure, a common metric in logistic regression that assesses model fit against actual data. The model coefficients and feature names corresponding to the optimal *λ* value were extracted, highlighting nonzero coefficients and their associated features (genes).

### Mendelian colocalization analysis

Bayesian colocalization analysis was used to evaluate the likelihood that 2 traits share common causal variants. This analysis examines whether the phenotypes share common genetic causal variants within the genetic regions of their corresponding expression quantitative trait locus genes. We defined the colocalization evidence threshold as PP.H4.abf > 80% and visualized the results using the stack_assoc_plot function from the gassocplot2 package.

### ST analysis

The cellular composition on the 10x Visium slides was assessed through a deconvolution analysis that integrates ST with scRNA-seq data specific to cancer types. These criteria were guided by the existing literature to ensure accuracy. The average expression levels of the top 25 marker genes for each cell type were calculated, generating a signature score matrix. The Cottrazm package was used to generate an enrichment score matrix, which facilitated further cellular composition analysis. A score of 1 defined the malignant group, 0 indicated normal, and other scores were classified as mixed.

Correlation visualization was performed using the linkET package. The AUCell package quantifies the expression enrichment of specific gene sets in each microregion using the AUC. AUC scores allow for comparative analysis of gene set expression across microregions, helping identify regions with active gene sets, such as specific gene tags or modules. AUC score visualization across microregions was performed.

### Pan-cancer single-cell analysis

Single-cell resolution gene expression data for pan-cancer were obtained from the TISCH database. The pheatmap package was used to generate heatmaps of the gene expression landscape. Using Euclidean distance as a metric and Ward’s minimum variance hierarchical clustering, patterns and trends in the data were discerned, aiding in the identification of conserved gene expression sources.

### The landscape of gene expression in pan-tumor from HPA and GTEx projects

Transcriptomic data from the HPA and GTEx projects were used to obtain consensus gene expression data for RNA across 50 tissues, summarizing the expression levels of consensus transcripts for each gene. The normalized expression values (nTPM) were calculated as the maximum nTPM value for each gene across both data sources. This dataset was derived from HPA version 23.0 and Ensembl version 109. Visualization was carried out using lollipop plots.

### Pan-cancer immune subtypes were different in *TFF3* gene high- and low-expression groups

In the influential study The Immune Landscape of Cancer, the authors conducted a large-scale immunogenomic analysis using over 10,000 tumor samples from TCGA across 33 cancer types. The cross-tumor investigation confirmed 6 distinct immune subtypes: C1 (wound healing), C2 (IFN-γ dominant), C3 (inflammatory), C4 (lymphocyte depleted), C5 (immunologically quiet), and C6 (transforming growth factor-β dominant).

### The genomics of drug sensitivity in cancer

The GDSC database includes 987 cell lines and 367 compounds. The R package oncoPredict was used to compile data files from these datasets [46]. A negative correlation indicates that higher gene expression levels increase drug sensitivity, while a positive correlation suggests greater drug resistance as gene expression increases.

### Differences and correlations in the IC_50_ values of drug sensitivity between high- and low-expression *TFF3* gene groups

The pRRophetic R package predicts clinical chemotherapy responses based on gene expression levels. This package is based on research by Paul Geeleher, Nancy Cox, and R. Stephanie Huang from the University of Minnesota [47]. The algorithm uses ridge regression to relate baseline gene expression levels to drug sensitivities observed in vitro. Using data from the Cancer Genomics Program, it estimates the IC_50_ for TCGA patients, enabling predictions of chemotherapy outcomes.

### MeTIL score

To evaluate the distribution of tumor-infiltrating lymphocytes using MeTIL, individual methylation values of MeTIL markers are converted into MeTIL scores through principal component analysis. MeTIL captures the infiltration levels of T cells, NK cells, B cells, and T regulatory cells and also reflects cytotoxic T lymphocyte functionality [48].

### EaSIeR analysis: CYT, IFN-γ, and T-cell-inflamed scores

EaSIeR is a tool that predicts biomarker-based immunotherapies by using cancer-specific immune response models to forecast anti-tumor immune responses based on RNA-seq data. The biomarker model has been validated experimentally in the literature, and the predictive performance of EaSIeR was confirmed using independent datasets from patients with 4 cancer types treated with anti-PD-1 or anti-PD-L1 therapies. This analysis includes 3 scores: CYT, IFN-γ, and T-cell-inflamed scores [49].

### The radiological datasets and participants

Radiological and clinical data for study subjects were obtained from The Cancer Imaging Archive for both the Duke and TCGA cohorts [50]. Transcriptomic data for TCGA cohort samples were obtained from the TCGA repository. Inclusion criteria specify that subjects must be female and have undergone preoperative contrast-enhanced MRI scans of the primary tumor site. Exclusion criteria include distant metastasis, receipt of neoadjuvant therapy, significant data gaps, poor image quality or absence of a discernible tumor, and lack of transcriptomic data in the TCGA database.

### Radiomics data preprocessing

The MRI images processing details were the same as in our previous study [5].

### Construction of an end-to-end deep learning model for ALNM prediction

We built an end-to-end deep learning model for ALNM prediction by improving the model training through dataset augmentation with the largest region of interest cross-sections and adjacent slices at ±2 and ±4 levels. The Shufflenet_v2_x1_0 model was trained using the Duke dataset and validated on the TCGA dataset. Resampling techniques were employed to address label imbalance. Training was conducted using the Adam optimizer with a learning rate schedule to ensure convergence. Model selection was based on accuracy to identify the model best suited for the prediction task.

### ALNM prediction based on the XGBoost algorithm combined with radiomics risk and *TFF3* expression levels

For ALNM prediction, we combined the XGBoost algorithm with radiomics risk and *TFF3* expression levels, integrating both imaging and genetic data to comprehensively assess the ALNM risk. Previous studies have shown that *TFF3* is a key gene affecting ALNM and survival prognosis, so we combined it with radiomics risk and modeled it using the XGBoost algorithm. To reduce the impact of redundant information on the XGBoost model, we first assessed the linear and nonlinear correlations between radiomics risk and *TFF3* expression using Pearson correlation coefficients and restricted cubic spline curves. The TCGA cohort was randomly divided into training and test sets at a 7:3 ratio. *TFF3* expression levels were normalized to a 0 to 1 range to align with the scale of radiomics prediction probabilities. We defined a hyperparameter space and used the Optuna tool for automated optimization, with AUC as the optimization metric. Finally, the Youden index was calculated to determine the optimal threshold for stratifying samples into risk groups within the radiogenomics model.

### Biological validation of radiogenomics risk stratification

We applied the limma method to identify DEGs across distinct risk groups. We then retrieved the h.all.v7.4.symbols.gmt gene set from MSigDB for comprehensive genomic analysis [51]. These gene sets served as a reference for mapping the DEGs. Using the R package clusterProfiler (version 3.14.3), we conducted an enrichment analysis to determine the biological significance of the genes, identifying pathways and processes associated with risk group variations.

### Cell lines and cell culture

Cell lines, including MCF-10A, MDA-MB-231, and MDA-MB-468, were provided by the Chinese Academy of Sciences Cell Bank in Shanghai. MCF-10A cells were cultured in Special Medium for MCF-10A cells (Procell, China), while MDA-MB-231 and MDA-MB-468 cells were cultured in Dulbecco’s modified Eagle medium (Gibco, China) supplemented with 10% fetal bovine serum (Cellmax, SA211.02, Beijing) at 37 °C in a 5% CO_2_ atmosphere.

### siRNA and cell transfection

siRNAs were synthesized by GenePharma (Suzhou, China). Cells were plated into 6-well plates at a density of 1 × 10^5^ cells per well and incubated until they reached 50% to 60% confluence. Subsequently, the cells were transfected with siRNA at a 1:2 ratio using Lipofectamine RNAiMAX Reagent (Thermo, USA) for 24 h. After 24 h, the medium was replaced with complete culture medium, and cells were cultured for an additional 24 h. To achieve overexpression, we purchased PCDH-*TFF3* from GenePharma (Suzhou, China) and transfected the cells with DNA using the jetPRIME transfection reagent (Polyplus, USA) at a 2:1 ratio, when cells were 60% to 80% confluent. After transfection, the medium was replaced 6 h later, and cells were cultured in complete medium for 48 h.

### Extraction of RNA and qRT-PCR

Total RNA was extracted from cells using TRIzol reagent (Absin, China). qRT-PCR was conducted using Vazyme ChamQ Universal SYBR qPCR Master Mix, and the results were analyzed by the 2^−ΔΔCT^ method. Relative expression levels were presented, with β-actin used as an internal control. All experiments were performed in triplicate.

### Western blot

Total protein extracts were separated using 10%/15% sodium dodecyl sulfate–polyacrylamide gel electrophoresis. The electrophoretic gel was transferred to a polyvinylidene difluoride membrane (0.45 μm, Millipore, USA) and blocked for 2 h with 5% nonfat dry milk. Primary antibodies were incubated overnight at 4 °C on the membranes. The antibodies used were rabbit anti-glyceraldehyde-3-phosphate dehydrogenase (1:5,000, Proteintech, China), rabbit anti-TFF3 (1:500, HUABIO, China), rabbit anti-MMP2 (1:1,000, Proteintech, China), rabbit anti-N-cadherin (1:1,000, Zenbio, China), rabbit anti-ERK1/2 (1:2,000, Proteintech, China), rabbit anti-p-ERK1/2 (1:1,000, Proteintech, China), rabbit anti-p38 (1:1,000, Proteintech, China), rabbit anti-p-p38 (1:1,000, Proteintech, China), mouse anti-JNK (1:2,000, Proteintech, China), and rabbit anti-p-JNK1/2/3 (1:500, Cohesion, China). The membranes were incubated with a secondary antibody conjugated to horseradish peroxidase at room temperature the following day. The membrane was washed 3 times, each for 10 min. Image acquisition was performed.

### Cell Counting Kit-8

Cells (2,000 per well) were seeded into 100 μl of medium on 96-well plates for the CCK-8 assay. Every 24 h, 10 μl of CCK-8 reagent (APExBIO, Houston, USA) was added to the medium, followed by a 2-h incubation at 37 °C. Absorbance was then measured at 450 nm.

### Colony formation assay

A single-cell suspension (1 × 10^3^ cells per well) was seeded into 6 wells and maintained at 37 °C with 5% CO_2_ for 10 to 14 d. The medium was replaced every 3 d. Finally, cells were fixed with 4% paraformaldehyde for 1 h, stained with 0.1% crystal violet for 30 min, and washed 3 times with PBS. The number of visible colonies was counted using ImageJ.

### 5-Ethynyl-2′-deoxyuridine

Cell proliferation was measured via the EdU Cell Proliferation Kit with Alexa Fluor 555 (CX003, CellorLab, China). On day 1, cells at 50% confluence were seeded onto a 24-well plate, and EdU labeling was performed on day 2. EdU-incorporated cells and DNA staining were imaged using a confocal microscope (ZEISS LSM 900, Germany). For statistical analysis, ImageJ was used to count the total number of cells and EdU-positive cells, and the proportion of EdU-positive cells was calculated.

### Transwell assay

Transwell chambers (Corning, USA) were used to evaluate cell migration. Cells were seeded into 600 μl of medium containing 20% serum in the lower chamber. After incubation for 72 h (MDA-MB-468) and 48 h (MDA-MB-231), cells were fixed with 4% paraformaldehyde for 1 h, followed by staining with 0.1% crystal violet for 30 min. Finally, migrating cells were counted using the ImageJ software.

### HLEC tube formation assay

HLECs were cultured for 8 h in 96-well plates containing culture medium. Tube formation was observed and photographed using a microscope. The number of tubes formed was quantified to assess tube formation.

### Immunohistochemistry

Tissue sections were deparaffinized and rehydrated, followed by incubation with 3% H_2_O_2_. Blocking was performed using bovine serum albumin (Solarbio, China) for 30 min. Tissue sections were incubated overnight at 4 °C with rabbit anti-TFF3 (1:400, Proteintech, China) and rabbit anti-Ki67 (1:400, Servicebio, China). The tissue sections were then incubated for 50 min with horseradish peroxidase-conjugated goat anti-goat secondary antibodies at 37 °C. 3,3′-Diaminobenzidine color development was performed, followed by nuclear restaining, dehydration, and sealing. Finally, images were scanned using Pannoramic MIDI (3DHISTECH, Hungary) and analyzed with CaseViewer.

### Hematoxylin and eosin

First, tissue samples were dewaxed and hydrated, followed by treatment with HE constant staining pretreatment solution for 1 min. HE staining was then performed, followed by dehydration and sealing. Images were scanned using Pannoramic MIDI (3DHISTECH, Hungary) and analyzed with CaseViewer.

### Xenograft mouse model

This study used female BALB/c-nu mice (4 to 5 weeks old, 13 to 15 g), provided by Hongzhou Ziyuan Laboratory Animal Technology Co., Ltd. In the tumorigenicity assay, mice were randomly assigned to the scramble, sh-*TFF3*#1, and sh-*TFF3*#2 groups. After a 1-week adaptation period, each mouse was injected with 5 × 10^6^ cells into the armpits. After 1 month, the animals were euthanized, and their weights and tumor volumes were measured following tumor resection. Tumor volume was measured via the formula *L* × *W*^2^/2. To evaluate the effects of *TFF3* knockdown on breast tumor metastasis, another animal experiment was conducted. Mice were randomly assigned to the scramble, sh-*TFF3*#1, and sh-*TFF3*#2 groups, and each mouse was injected with 4 × 10^6^ cells in 40 μl of PBS into the footpad. After 2 months, the tumors were removed from the footpad and enlarged LNs, and their sizes were measured.

### Statistics and repeatability

Unpaired, 2-tailed Wilcoxon rank-sum tests were conducted to compare cell distributions between the 2 groups. Paired, 2-tailed Wilcoxon rank-sum tests were applied to compare cell distributions between matched ALNM+ PBC and ALNM− PBC groups. Statistical computations and presentations were conducted via R. A significance level of *P* < 0.05 was applied.

## Ethical Approval

This study involving human participants was reviewed and approved by the Ethics Committee of the hospital (Approval Number: FSYYY-EC-SOP-008-02.0-A09). Written informed consent was obtained from all subjects. Animal experiments complied with the ARRIVE guidelines and were approved by the Institutional Animal Care and Use Committee (Approval Number: RYE2023102402).

## Data Availability

The data used in this study can be obtained by request to Jianguo Lai (laijianguo@gdph.org.cn).
