# Supplementary material for: Lymph Node Metastasis-Associated Spatiotemporal Mapping of the TFF3-Linked Niche in Breast Cancer: Integrating Radiogenomic Signatures with Immune-Ecosystem Remodeling
Source: Research (Wash D C). 2026 Jan 15;9:1016. doi: 10.34133/research.1016 (PMC12805538; doi:10.34133/research.1016)
Supplement: Supplementary 1 — Figs. S1 to S10 Table S1 [file research.1016.f1.pdf]

Supplementary Material

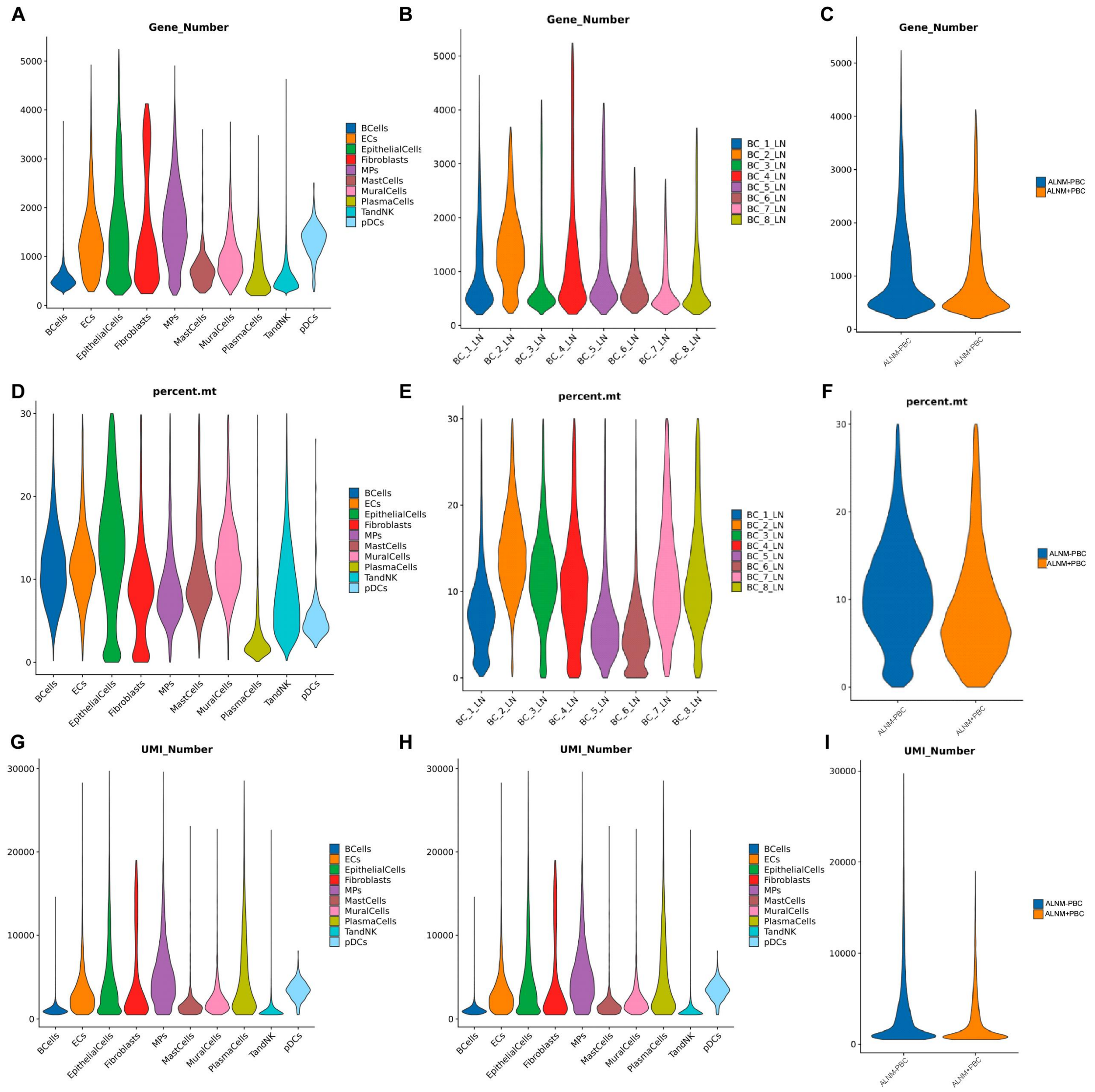

Figure S1. Quality control of scRNA-seq. (A, D, G) Violin plots showing the distribution of Gene_Number, percent.mito, and UMI_Number across different cell clusters. **(B, E, H)** Violin plots displaying the distribution of Gene_Number, percent.mito, and UMI_Number across each sample. **(C, F, I)** Violin plots illustrating the distribution of Gene_Number, percent.mito, and UMI_Number in ALNM+ and ALNM-PBC.

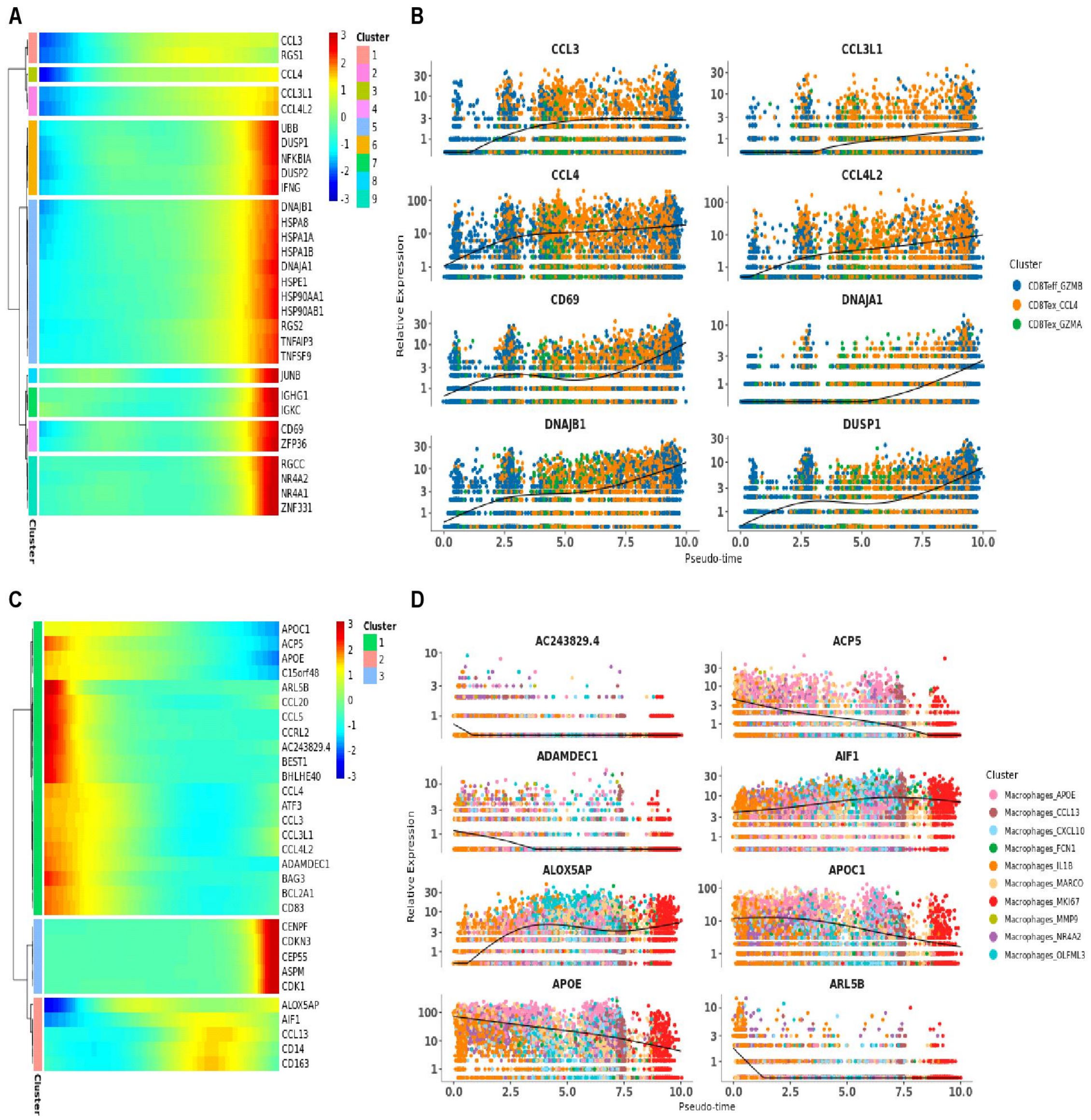

Figure S2. Characterization of CD8+ T cells and macrophages in the tumor microenvironment.

(A) Expression of the most variable genes involved in the CD8+ T cell state transition. **(B)** Monocle pseudo-time trajectory analysis of each CD8+ T cell subcluster with marker genes. **(C)** Expression of the most variable genes involved in the macrophage state transition. **(D)** Monocle pseudo-time trajectory analysis of each macrophage subcluster with marker genes.

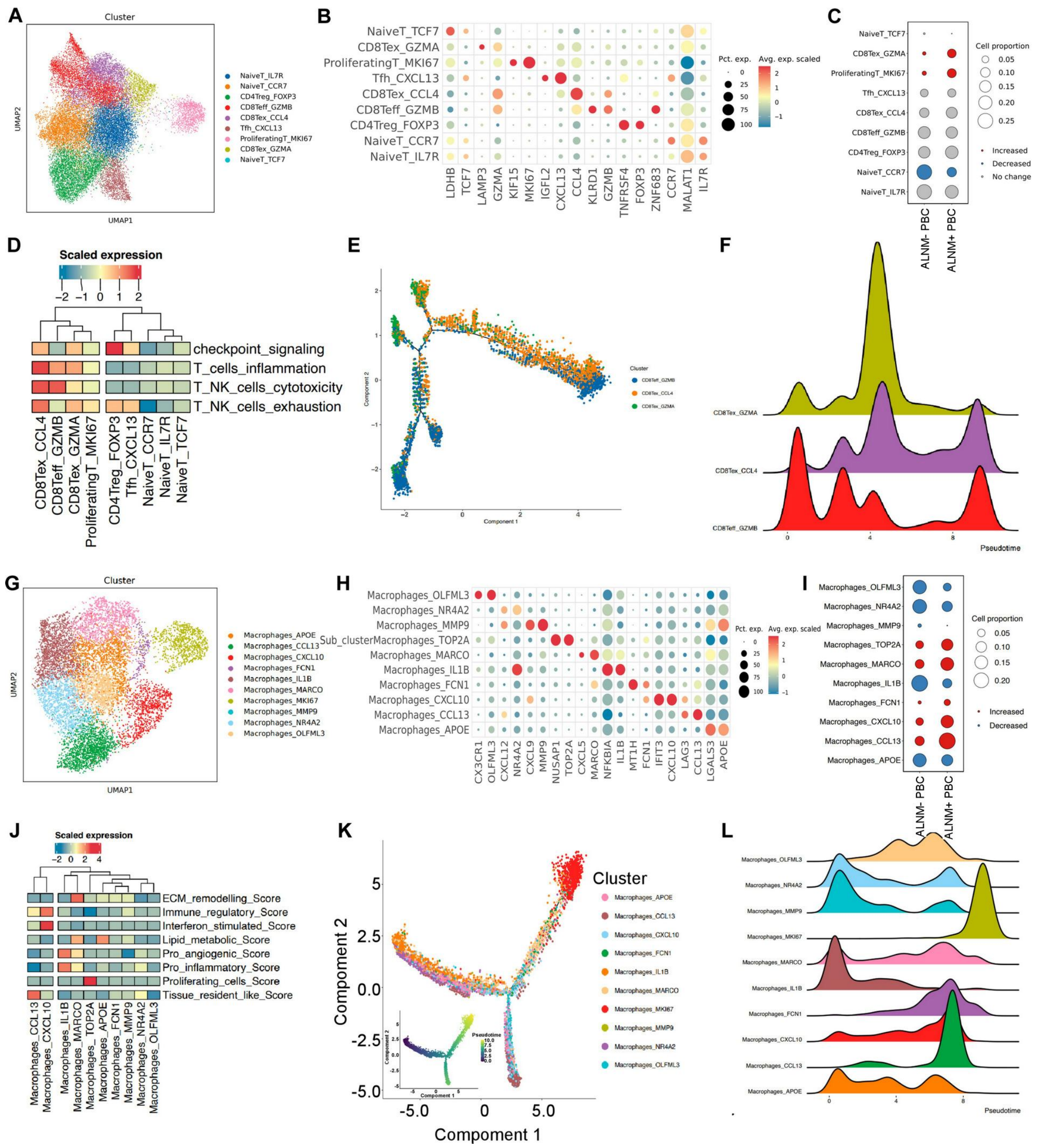

Figure S3. Characterization of dendritic cells in the tumor microenvironment. (A) Reclustering of dendritic cells and visualization of the profile of each subtype using a UMAP plot. **(B)** Dot plot showing the expression levels of canonical marker genes in each dendritic cell subcluster. **(C)** Proportion of each dendritic cell subcluster in groups with or without ALNM. **(D)** Gene signature scores of each dendritic cell cluster were analyzed and quantified. **(E)** Monocle pseudo-time trajectory analysis of dendritic cells with highly variable gene expression. Each dot on the pseudo-time curve represents a single cell, colored according to its cluster label. **(F)** Expression of the most variable genes involved in dendritic cell state transitions. **(G)** Monocle pseudo-time trajectory analysis of each dendritic cell subcluster with marker genes.

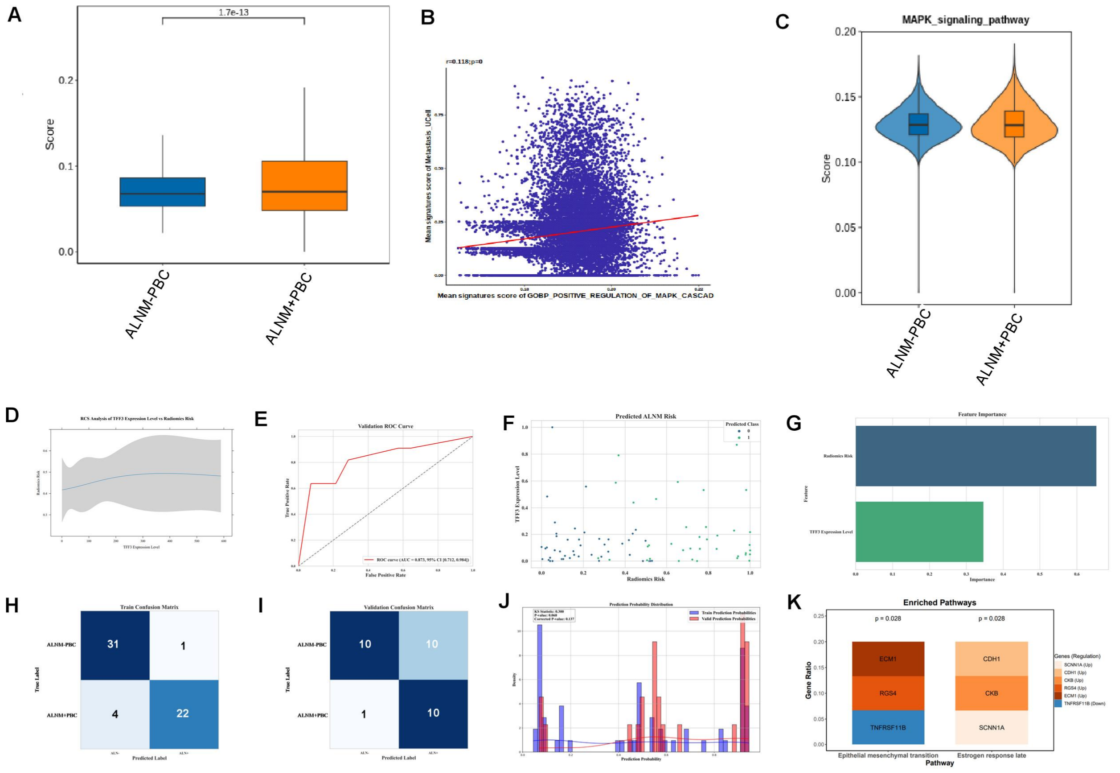

Figure S4. Joint prediction of machine learning based on radiomics feature and *TFF3* gene expression. **(A)** Bar plot of the EMT score for tumor cells in PBC with or without ALNM. **(B)** Scatter plot showing the correlation between the metastasis program and the MAPK cascade in ALNM+ and ALNM-PBC. **(C)** Violin plot of the MAPK cascade in tumor cells from PBC with or without ALNM. **(D)** RCS curve showing the nonlinear relationship between *TFF3* expression levels and radiomics risk. **(E)** Distribution of *TFF3* expression levels and radiomics risk across samples with different prediction classifications. **(F-G)** ROC curves predicting ALNM in training and testing sets using radiogenomics models. **(H-I)** Confusion matrix of the radiogenomics model based on the optimal classification threshold in training and test sets. **(J)** The histogram with kernel density estimation shows the density distribution of prediction probabilities in the training and testing datasets. **(K)** The Kolmogorov-Smirnov (KS) statistic and p-value indicate the difference between the distributions of two datasets. The feature importance histogram shows the significance of *TFF3* expression levels and radiomics risk in predicting ALNM in the radiogenomics model.

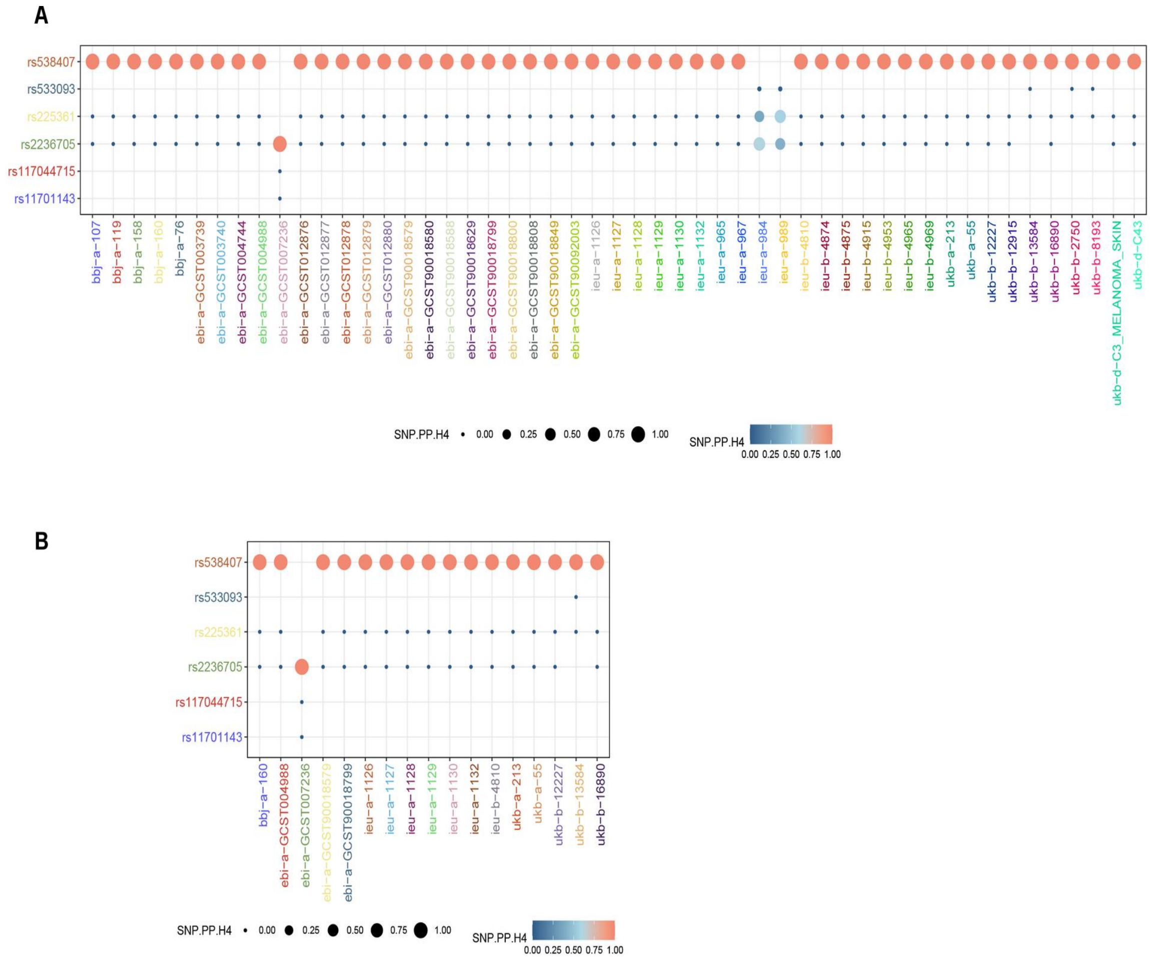

Figure S5. eQTL-GWAS colocalization visualized using the gassocplot2 package. (A-B) Stacked regional association plots display overlapping genomic regions for eQTLs and GWAS traits based on GRCh37/hg19 coordinates. Panel shows pan-cancer results **(A)**, while panel focuses on breast cancer **(B)**. Dots represent SNPs with the minimum P-values for eQTL and GWAS signals, respectively. The cutoff for colocalization evidence was set at PP.H4.abf > 0.75.

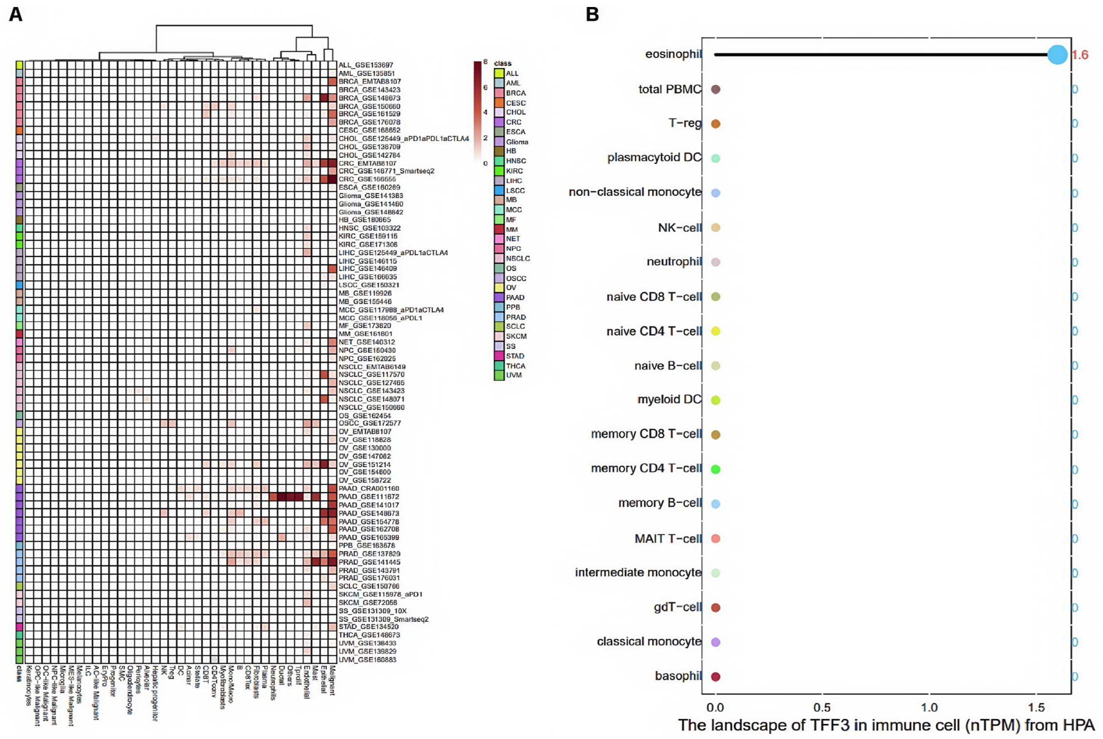

Figure S6. Expression of the *TFF3* gene in pan-cancer single-cell subclusters and various immune cells. (A) Single-cell resolution gene expression data from pan-cancer were retrieved from the TISCH database. The pheatmap package was used to generate heatmaps depicting the gene expression landscape. **(B)** The landscape of *TFF3* expression in various immune cells from the Human Protein Atlas (HPA) database and Genotype-Tissue Expression (GTEx) project. The y-axis represents various tissues, while the x-axis denotes gene expression levels (nTPM). The positions of the points in the lollipop plot represent gene expression levels across different tissues.

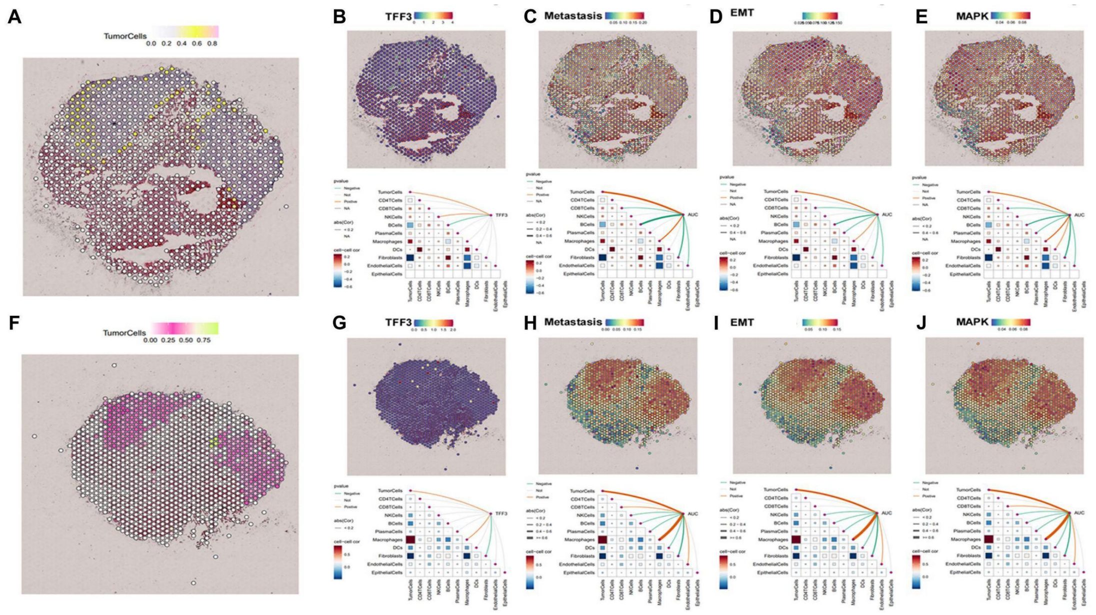

Figure S7. Decoding spatial transcriptomes focusing on *TFF3*, MAPK, and EMT scores in PBC. (A-J) Spatial mapping of transcriptomes focusing on *TFF3*, metastasis expression program, MAPK cascade, and EMT scores in PBC. The Spatial Feature Plot function in the Seurat package was used to visualize the gene expression landscape in each microregion. Spearman correlation analysis was performed to assess the associations between cellular content and gene expression levels or gene scores at all spots. These correlations were visualized using the linkET package.

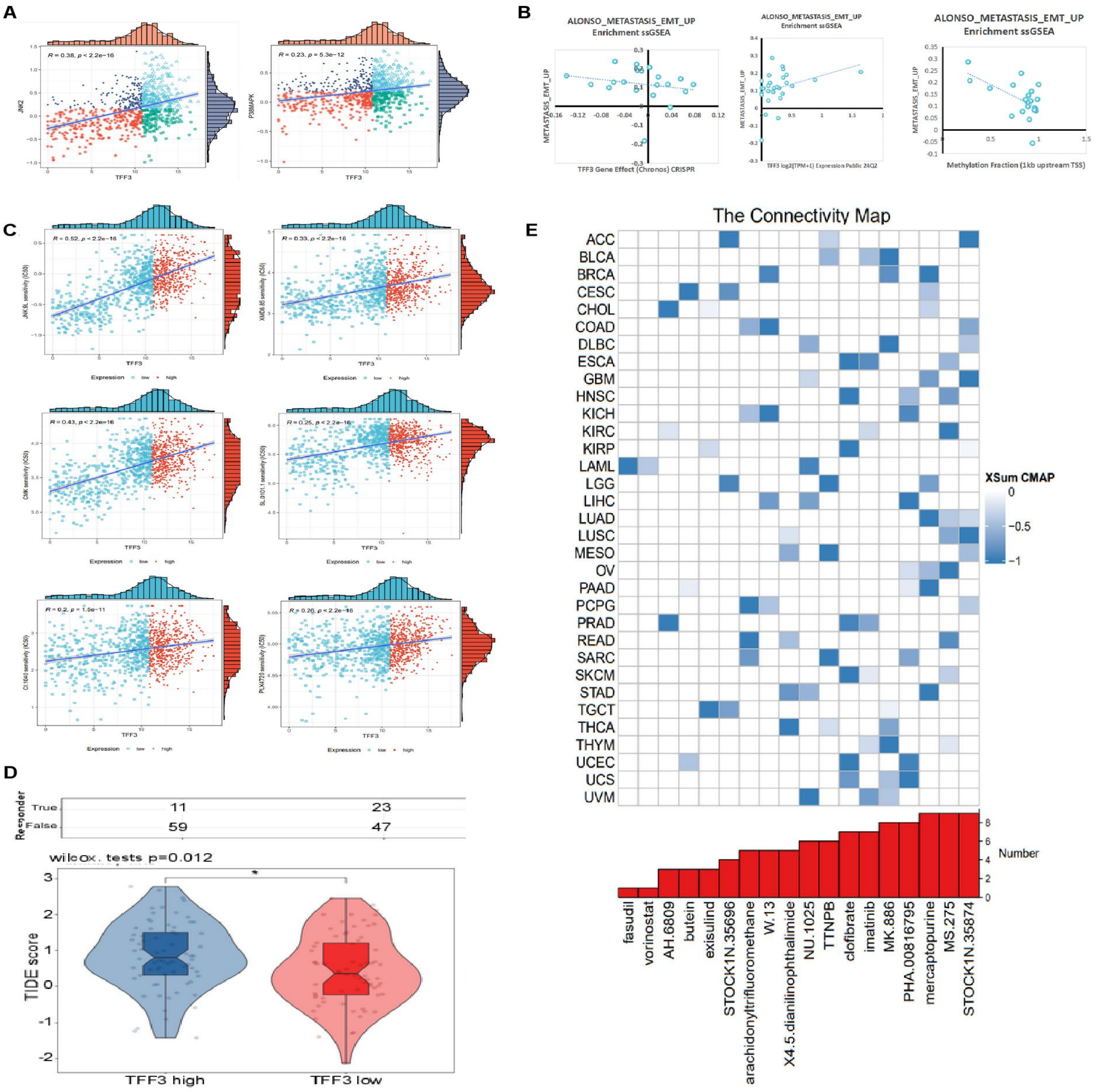

Figure S8. Comprehensive Analysis of *TFF3* in Breast Cancer: Multi-modal Correlations and Therapeutic Implications. (A) The scatter plot showed a correlation between *TFF3* protein expression and key proteins of the MAPK pathway. **(B)** The correlation between *TFF3* gene expression, CRISPR gene editing, methylation, and the metastasis-EMT-up pathway in various PBC cell lines. **(C)** Scatter plots illustrated the Spearman correlation between *TFF3* gene expression and the IC50 values of various MAPK pathway inhibitors, including JNK9L, CMK, XMD8.85, PLX4720, SL.0101.1, and CI.1040. **(D)** TIDE analysis was performed to assess the efficacy of immune checkpoint blockade (ICB) therapy in *TFF3* high- and low-expression groups in breast cancer (BRCA). Upper panel: A statistical overview of immune responses across different groups, as predicted by our model. Bottom panel: Visualization of immune response

score distributions across groups. The Wilcoxon test was used to determine statistical significance between the two groups. **(E)** The XSum algorithm identified potential small molecules and drugs that could correct biological effects caused by dysregulated *TFF3* gene expression in pan-cancer based on data from the cMAP database. Each scatter point represents a distinct compound, with the y-axis showing similarity scores for 1288 compounds, derived by comparing gene-related features using the XSum (eXtreme Sum) method. Compounds with lower scores may inhibit gene-mediated oncogenic effects.

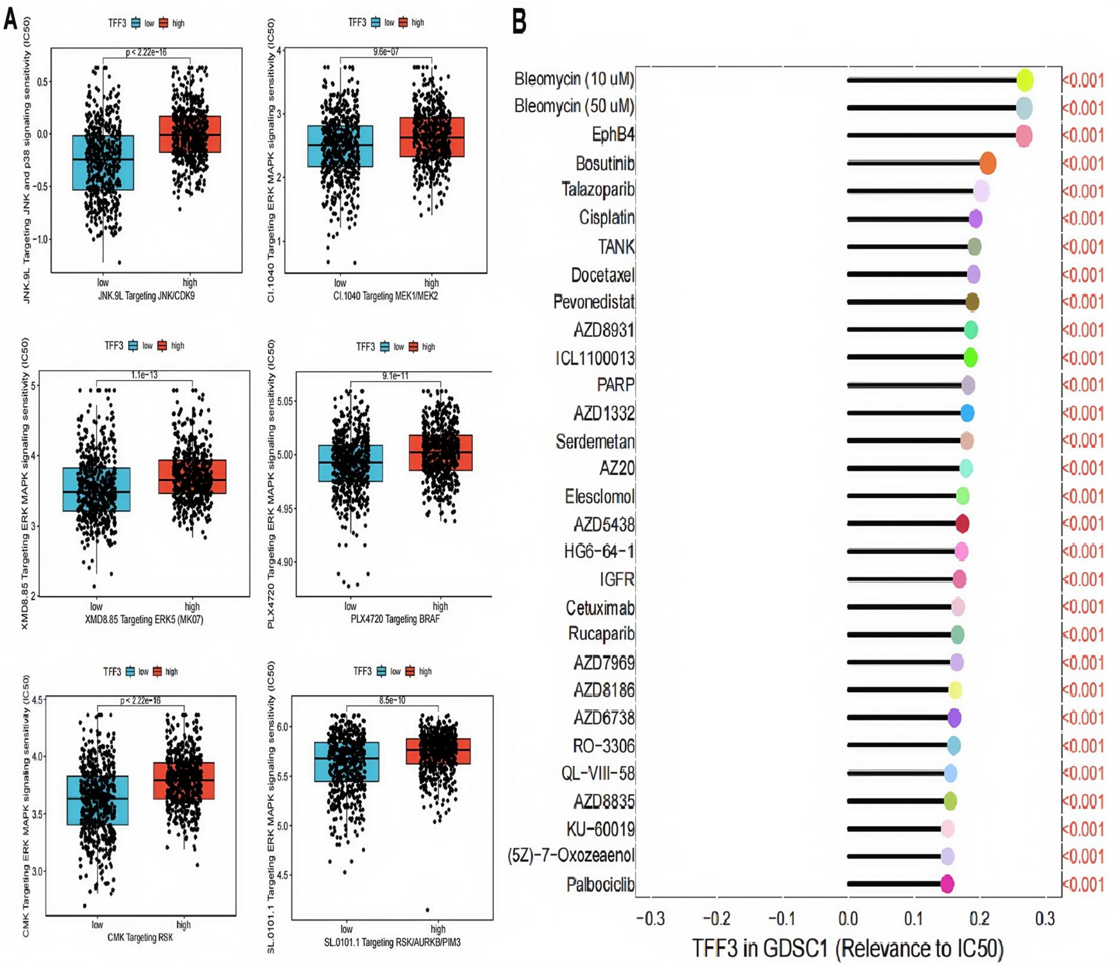

Figure S9. ***TFF3*** **gene expression correlates with MAPK pathway inhibitors and anticancer compounds in the GDSC1 database. (A)** Disparities in drug sensitivity, measured by IC50 values, are observed among various MAPK pathway inhibitors (including JNK9L, CMK, XMD8.85, PLX4720, SL.0101.1, and CI.1040) between groups with high and low *TFF3* gene expression levels. **(B)** Spearman correlation between *TFF3* gene expression and the IC50 or AUC values of drugs in the GDSC1 database.

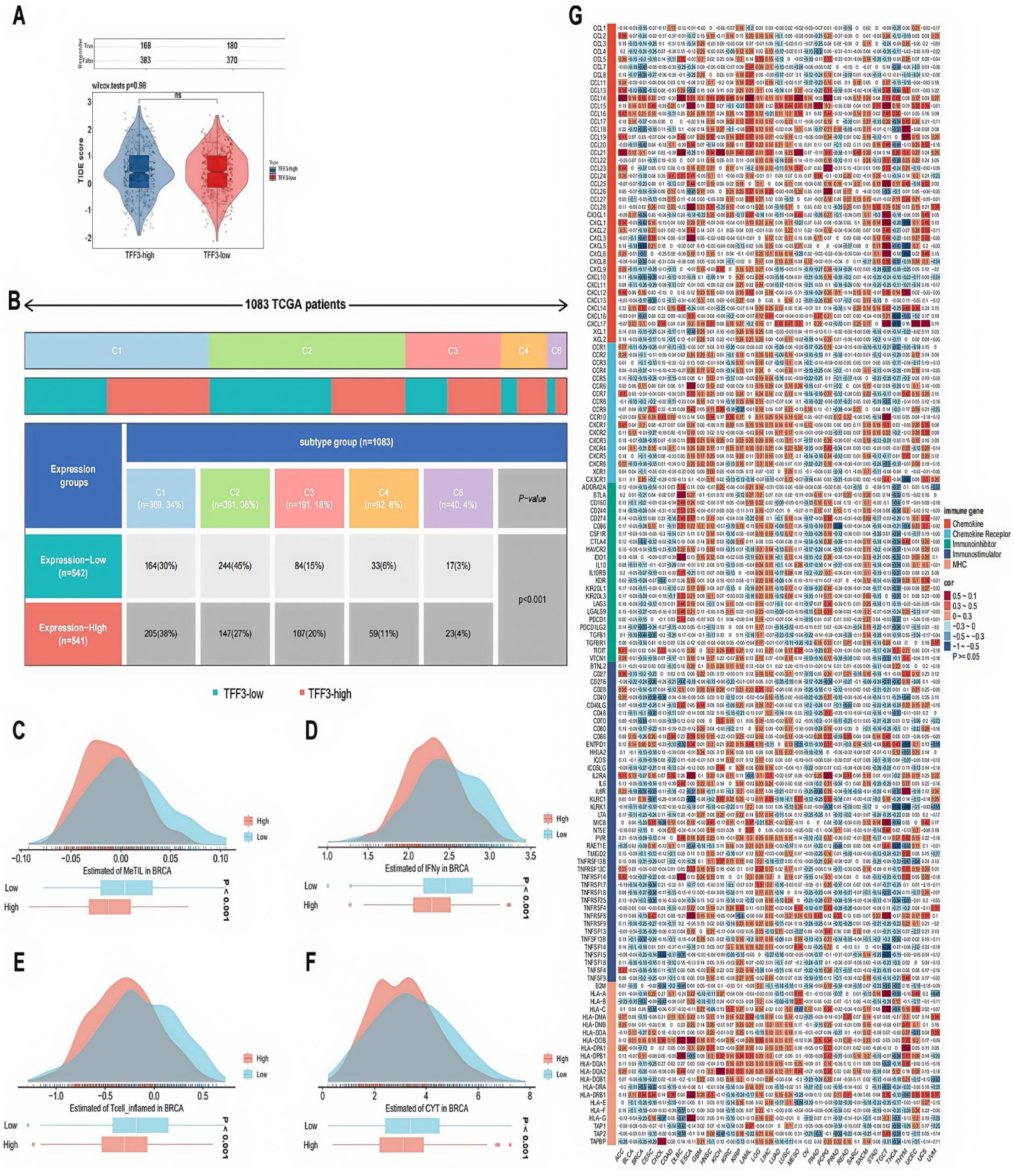

Figure S10. Association between *TFF3* gene expression and immunoscore. (A) TIDE analysis of immune checkpoint blockade (ICB) therapy efficacy in *TFF3* high- and low-expression groups in PBC. Upper panel: Statistical overview of immune responses across groups as predicted by our model. Lower panel: Visualization of immune response score distributions across different groups. The Wilcoxon test was used to assess significance between the two groups. **(B)** In a pan-cancer framework, six immune subtypes are defined by divergent gene expression patterns in *TFF3* high- and low-expression groups. **(C-F)** Disparities in MeTIL score **(C)**, INFγ score **(D)**, Tcell_inflamed score **(E)**, and CYT score **(F)** between *TFF3* high- and low-expression groups. **(G)** Pearson correlation analysis shows the relationship between *TFF3* expression and immune-related genes

across pan-cancer datasets. The heatmap displays tumors along the horizontal axis, with the vertical axis categorizing five types of immune-related genes: chemokine-related genes, chemokine receptor-related genes, immunoinhibitor genes, immunostimulator genes, and major histocompatibility complex (MHC) genes.

Table S1 Baseline characteristics of the Duke and TCGA cohort

Variables	Duke cohort	TCGA cohort
No. of patients	313(100)	83(100)
Age	54.81±10.47	54.90±10.61
T stage		
T1	166(53.0)	29(34.9)
T2	129(41.2)	50(60.2)
T3/T4	18(5.8)	4(4.9)
N stage		
N0	223(71.2)	45(54.2)
N1-3	90(28.8)	38(45.8)
